# Tissue immunoexpression of IL-6 and IL-18 in aging men with BPH and MetS and their relationship with lipid parameters and gut microbiota-derived short chain fatty acids

**DOI:** 10.18632/aging.205091

**Published:** 2023-10-16

**Authors:** Weronika Ratajczak, Maria Laszczyńska, Aleksandra Rył, Barbara Dołęgowska, Olimpia Sipak, Ewa Stachowska, Marcin Słojewski, Anna Lubkowska

**Affiliations:** 1 Department of Functional Diagnostics and Physical Medicine, Pomeranian Medical University, Żołnierska, Szczecin 71-210, Poland; 2 Department of Nursing, State University of Applied Sciences, Leśna, Koszalin 75-582, Poland; 3 Department of Medical Rehabilitation and Clinical Physiotherapy, Pomeranian Medical University, Żołnierska, Szczecin 71-210, Poland; 4 Department of Laboratory Medicine, Pomeranian Medical University, Powstańców Wielkopolskich, Szczecin 70-111, Poland; 5 Department of Obstetrics and Pathology of Pregnancy, Pomeranian Medical University, Żołnierska, Żołnierska, Szczecin 71-210, Poland; 6 Department of Human Nutrition and Metabolomics, Pomeranian Medical University, Broniewskiego, Szczecin 71-460, Poland; 7 Department of Urology and Urological Oncology, Pomeranian Medical University, Powstańców Wielkopolskich, Szczecin 70-111, Poland

**Keywords:** benign prostatic hyperplasia (BPH), metabolic syndrome (MetS), lipids, interleukin 6 (IL-6), interleukin 18 (IL-18), short-chain fatty acids

## Abstract

Recent studies indicate that inflammation is one of the causes of the development of benign prostatic hyperplasia (BPH). Inflammation may result from past infections, metabolic disorders, but also from the state of functioning of the intestinal microbiota. The aim of the study was to assess whether the diagnostic lipid parameters for metabolic syndrome and short-chain fatty acids (SCFAs) are related to the immunoexpression of interleukins in prostate tissue with benign hyperplasia.

The study involved 103 men with BPH, who were divided into two groups depending on the presence of MetS. We analysed tissue immunoexpression of two proinflammatory interleukins: IL-6, which is known to be involved in the development of BPH, and IL-18, which has not been analysed so far. The results of our study indicate that men with BPH + MetS in the stroma of the prostate have a significantly higher overall percentage of IL-6+ cells compared to men without MetS (*p* = 0.034). The analysis of IL-18 immunoexpression in prostate tissue indicated that in men with BPH + MetS, the glandular part of the prostate had a significantly higher percentage of cells with strong IL-18 expression (*p* = 0.040). We also noticed a relationship between tissue expression of IL-6 and IL-18 and lipid parameters (TG and HDL). We conclude that lipid disorders occurring in men with BPH increase inflammation in the prostate gland. Moreover, it has also been demonstrated for the first time that, indirectly, through SCFAs, the gut microbiota can act to prevent or create an inflammatory microenvironment in the prostate gland.

## INTRODUCTION

Benign prostatic hyperplasia (BPH) is one of the most commonly diagnosed urological diseases in men over 50 years of age. BPH is characterised by prostatic stromal cell proliferation, leading to prostatic bladder obstruction (BOO) and lower urinary tract symptoms (LUTS), which together reduce quality of life (QoL). The development of BPH is very often associated with the existence of comorbidities, such as diabetes, cardiovascular diseases, and even neurological diseases [1]. Many studies also indicate a relationship between the metabolic syndrome (MetS) and the risk of LUTS and BPH [2–4]. MetS is defined as the combination of obesity, dyslipidaemia, hyperglycaemia, high blood pressure and insulin resistance. The factor that contributes to the initiation of pathological changes in the prostate, and consequently its benign hyperplasia, is chronic inflammation resulting, among others, from metabolic disorders [5]. Moreover, MetS is accompanied by sex hormone disorders, which is also one of the etiological factors of BPH [6].

So far, the exact pathogenesis and mechanism of BPH progression have not been fully understood. It is known, however, that this is a multifactorial process, and in recent years there is more and more evidence that one of the causes of BPH is an immune reaction resulting from inflammation [7, 8]. Inflammation can be caused by microbial, bacterial or viral factors, hormonal changes, MetS, dietary factors, urinary regurgitation, as well as an autoimmune reaction [9].

Cells that form inflammatory infiltrates are CD3 T lymphocytes (70–80%), CD19 or CD20 B lymphocytes (10–15%) and macrophages (15%) [10, 11]. Activated cells secrete cytokines and growth factors that promote the proliferation of prostate stromal and epithelial cells [9]. A key factor in the development of BPH is pro-inflammatory interleukin 17 (IL-17), secreted by Th17 lymphocytes, and is also involved in the development of colon cancer, breast cancer, lung cancer, pancreatic cancer and prostate cancer [12]. IL-17 is a factor which stimulates epithelial and endothelial cells and fibroblasts to secrete other factors that also have a pro-inflammatory effect, such as IL-1β, TNF-α, IL-8, inducible nitric oxide synthase (iNOS) and cyclooxygenase-2 (COX-2) [13]. In addition, IL-17 is involved in the progression of BPH, among others through activation of the nuclear-factor-kappa-B (NF-κB) pathway, which leads to the secretion of other pro-inflammatory interleukins, e.g., IL-1, -6 and -8 [14].

Interleukin 6 is a pleiotropic cytokine and, depending on the context of the immune response, is considered an anti- or pro-inflammatory cytokine. IL-6 has a wide range of action, not only affects the cells of the immune system, but also has a hormone-like effect, thus significantly influencing the body’s homeostasis [15] and is an important factor involved in the process of haematopoiesis, bone metabolism and embryonic development [16]. Due to its biological properties, IL-6 is currently a significant therapeutic target for chronic inflammatory diseases, including autoimmune and neoplastic diseases [16, 17].

It has been confirmed in molecular and immunohistochemical studies that IL-6 in benign prostatic hyperplasia is a factor involved in paracrine and autocrine epithelial cell growth regulatory loop [14].

Interleukin 18 (IL-18), also called gamma interferon induction factor (IFN-γ), is a pleiotropic cytokine belonging to the interleukin 1 family [18]. The formation of an active and mature form of IL-18 depends on caspase-1 resulting from the assembly of protein complexes – inflammasomes, which are crucial for the initiation of sterile inflammation in metabolic disorders and chronic inflammatory diseases [19]. Interleukin 18 is secreted from monocytes/macrophages, however more than 80% of the precursor form of IL-18 remains in this form inside the cell. It can be secreted by various cells – immune (macrophages, Kupffer cells and dendritic cells) and cells that are not components of the immune system (including keratinocytes and tumour cells), and its role is key in many pathophysiological processes and diseases [18]. This cytokine is involved in processes related to hypertrophy, cell proliferation and fibrosis through various signalling pathways. IL-18 is also involved in enhancing the activity of NK (natural killer) cells (stimulating the secretion of INF-γ) and activating the proliferation of T lymphocytes [20]. Interleukin 18, only in the presence of IL-12 or IL-15, has the ability to induce IFN-γ. Among the diseases in which IL-18 plays a significant role is, i.e., systemic lupus, rheumatoid arthritis, type 1 diabetes, Crohn’s disease and psoriasis. In addition to its immunomodulatory effect, IL-18 is also characterised by a pro-inflammatory effect. Like other pro-inflammatory cytokines, it increases the amount of cellular adhesion molecules, increases the synthesis of nitric oxide and the production of chemokines [21]. It has also been noticed that elevated levels of IL-18 occur in people diagnosed with obesity, insulin resistance, hypertension and lipid disorders, which are components of the metabolic syndrome [22]. It has also been found that IL-18 is a specific biomarker of clinical symptoms of atherosclerosis in MetS patients [23]. However, in BPH, IL-18 acts as a factor stimulating the growth of prostate stromal cells [24].

The main aim of the study was to assess whether the diagnostic parameters for metabolic syndrome and short-chain fatty acids (SCFAs) are related to the immunoexpression of pro-inflammatory interleukins in prostate tissue with benign hyperplasia.

## RESULTS

### Baseline characteristic of BPH patients

The study group involved 103 patients with a diagnosis of BPH, qualified for transurethral resection of the prostate (TURP). Among the study group, the following were distinguished: patients with BPH (mean PV = 66.30 ml ± 30.73, mean Q_max_ = 9.45 ml/s ± 5.95), and patients with BPH + MetS (mean PV = 68.53 ml ± 23.38, mean Q_max_ = 11.48 ml/s ± 7.32).

There were no statistically significant differences in diagnostic parameters for BPH between the study groups (Table 1).

**Table 1:** Characteristics of patients diagnosed with BPH depending on the presence of MetS.

**Parameter**	**BPH without MetS (*n* = 61)**					**BPH with MetS (*n* = 42)**					***p*-value**
	**Mean**	**Median**	**Min**	**Max**	**SD**	**Mean**	**Median**	**Min**	**Max**	**SD**	
**Age (years)**	66.25	67	50	79	6.73	66.76	66.5	49	79	6.23	0.694
**PV (ml)**	66.3	60	35	240	30.73	68.53	65	40	120	23.38	0.694
**Q_max_ (ml/s)**	9.45	8.2	2	29.2	5.96	11.48	10.65	2.2	40	7.32	0.156
**IPSS**	20.02	21	4	34	7.07	19.42	20	3	35	8.79	0.708
**IPSS-S (storing)**	9.1	9	1	15	3.49	8.66	9	2	15	3.73	0.545
**IPSS-V (voiding)**	10.91	12	0	20	4.55	10.76	12	0	20	5.98	0.886
**QLS**	3.19	3	1	5	1.13	3.44	4	0	5	1.42	0.174

Abbreviations: BPH: benign prostatic hyperplasia; n: number; Min: minimum; Max: maximum; SD: standard deviation; PV: prostate volume; Q_max_: maximum flow rate in uroflowmetry; IPSS: International Prostate Symptom Score; QLS: Quality of Life score; p: statistical significance.

Anthropometric measurements were also performed on the patients (Table 2). A statistically significant difference was also found in the measurement of systolic (*p* = 0.004) and diastolic blood pressure (*p* = 0.039). Significantly higher values of these parameters were noted in the group of men with BPH + MetS.

**Table 2:** Anthropometric parameters and pressure measurement in patients diagnosed with BPH depending on the presence of MetS.

**Parameter**		**BPH without MetS (*n* = 61)**					**BPH with MetS (*n* = 42)**					***p*-value**
		**Mean**	**Median**	**Min**	**Max**	**SD**	**Mean**	**Median**	**Min**	**Max**	**SD**	
**Body weight (kg)**		81.15	80.00	55.00	113.00	11.91	90.17	89.00	65.00	116.00	12.57	0.694
**Height (m)**		1.74	1.75	1.60	1.87	0.06	1.74	1.75	1.63	1.90	0.06	0.694
**BMI (kg/m^2^)**		26.67	26.45	18.59	34.11	3.27	29.71	29.23	21.74	42.61	3.83	0.263
**WC (cm)**		96.69	97.00	72.00	121.00	11.21	107.39	106.00	90.00	136.00	9.72	0.337
**Blood pressure**	**systolic**	134.38	134.00	94.00	184.00	15.93	143.67	140.00	105.00	192.00	17.90	0.004^*^
	**diastolic**	80.29	80.00	60.00	101.00	8.64	85.00	83.50	67.00	108.00	8.63	0.039^*^

Abbreviations: BPH: benign prostatic hyperplasia; n: number; Min: minimum; Max: maximum; SD: standard deviation; WC: waist circumference; BMI: body mass index; p: statistical significance; ^*^: statistical significant parameter.

### Biochemical parameters and interleukin-6 and -18 in serum

Serum biochemical parameters and interleukins in the study groups were also measured (Table 3). The levels of TG (*p* = 0.005) were statistically significantly higher in patients with BPH + MetS, while levels of LDL cholesterol were statistically significantly higher in patients with BPH (*p* = 0.045).

**Table 3:** Biochemical parameters and interleukin -6 and -18 in patients diagnosed with BPH depending on the presence of MetS.

**Parameter**	**BPH without MetS (*n* = 61)**					**BPH with MetS (*n* = 42)**					***p*-value**
	**Mean**	**Median**	**Min**	**Max**	**SD**	**Mean**	**Median**	**Min**	**Max**	**SD**	
**TG (mmol/l)**	156.415	150.964	114.05	213.774	25.13	187.65	165.84	117.355	560.331	74.75	0.005^*^
**Cholesterol (mg/dl)**	208.334	203.717	143.494	404.096	40.821	199.363	188.129	153.16	304.437	34.398	0.178
**HDL (mg/dl)**	53.163	52.578	41.704	71.076	7.216	51.415	50.448	41.928	72.87	6.83	0.251
**LDL (mg/dl)**	124.555	116.372	70.871	321.069	40.198	110.418	99.835	41.042	222.812	33.974	0.045^*^
**TG/HDL ratio**	3.182	2.978	0.388	14.665	1.741	3.739	3.561	1.981	12.949	1.74	0.011^*^
**LDL/HDL ratio**	2.374	2.31	0.032	7.699	1.045	2.193	2.047	0.563	4.456	0.739	0.358
**Glucose (mg/dl)**	75.655	77.636	49.425	100.415	13.038	83.867	85.623	54.952	109.169	13.212	0.916
**IL-6 (pg/ml)**	5.141	3.864	1.136	13.03	2.988	4.37	2.955	1.061	13	3.08	0.182
**IL-18 (pg/ml)**	363.343	357.995	324.151	419.23	27.13	364.287	366.017	323.31	421.414	28.825	0.918

Abbreviations: BPH: benign prostatic hyperplasia; n: number; Min: minimum; Max: maximum; SD: standard deviation; TG: triglycerides; HDL: high density lipoprotein; LDL: low density lipoprotein; p: statistical significance; ^*^: statistical significant parameter.

There were no statistically significant differences in serum levels of IL-6 and IL-18 between the study groups (Table 3), which indicates that serum concentration of IL-6 and IL-18 are not good markers determining inflammation in patients with BPH + MetS. Moreover, the concentrations of IL-6 in the studied groups of patients are comparable to results obtained in healthy patients [25], the same applies to IL-18 [26].

### Stool SCFAs levels in BPH patients with regard to MetS

The study shows that the patients with BPH and with MetS only had significantly lower levels of stool isocaproic acid (*p* = 0.034) (Table 4). In addition, in our previous studies, we noticed significant differences in the levels of stool SCFAs between healthy patients and patients with BPH. The results have already been published [27].

**Table 4:** Short-chain fatty acids in patients diagnosed with BPH depending on the presence of MetS.

**SCFAs (%)**	**Patients with BPH *n* = 103**										***p*-value**
	**Without MetS (*n* = 61)**					**With MetS (*n* = 42)**					
	**Mean**	**Median**	**Min**	**Max**	**SD**	**Mean**	**Median**	**Min**	**Max**	**SD**	
**C2:0**	33.279	33.227	15.205	49.396	7.088	33.373	32.402	19.923	61.909	8.556	0.660
**C3:0**	20.088	19.412	0.903	35.717	5.784	21.175	20.031	4.921	36.581	7.000	0.562
**C4:0i**	4.838	4.723	0.370	16.163	2.281	4.489	4.413	0.438	9.394	2.154	0.492
**C4:0n**	22.759	23.054	6.094	39.532	8.039	23.880	24.232	6.596	47.467	8.945	0.457
**C5:0i**	9.911	9.554	1.944	33.867	4.948	8.902	8.842	0.450	19.674	5.050	0.336
**C5:0n**	6.321	6.443	0.655	16.167	2.670	5.826	6.312	0.795	12.820	2.611	0.403
**C6:0i**	0.217	0.167	0.023	0.7441	0.172	0.141	0.103	0.028	0.404	0.094	0.034^*^
**C6:0n**	2.242	1.578	0.088	10.709	7.253	1.943	0.822	0.123	5.911	1.955	0.388

Abbreviations: BPH: benign prostatic hyperplasia; MetS: metabolic syndrome; n: number; Min: minimum; Max: maximum; SD: standard deviation; SCFAs: short-chain fatty acids; C2:0: acetic acid; C3:0: propionic acid; C4:0i: isobutyric acid; C4:0n: butyric acid; C5:0i: isovaleric acid; C5:0n: valeric acid; C6:0i: isocaproic acid; C6:0n: caproic acid; p: statistical significance; ^*^: statistical significant parameter.

### Immunoexpression of interleukin-6 and -18 in the prostate tissue

**Figure 1:**
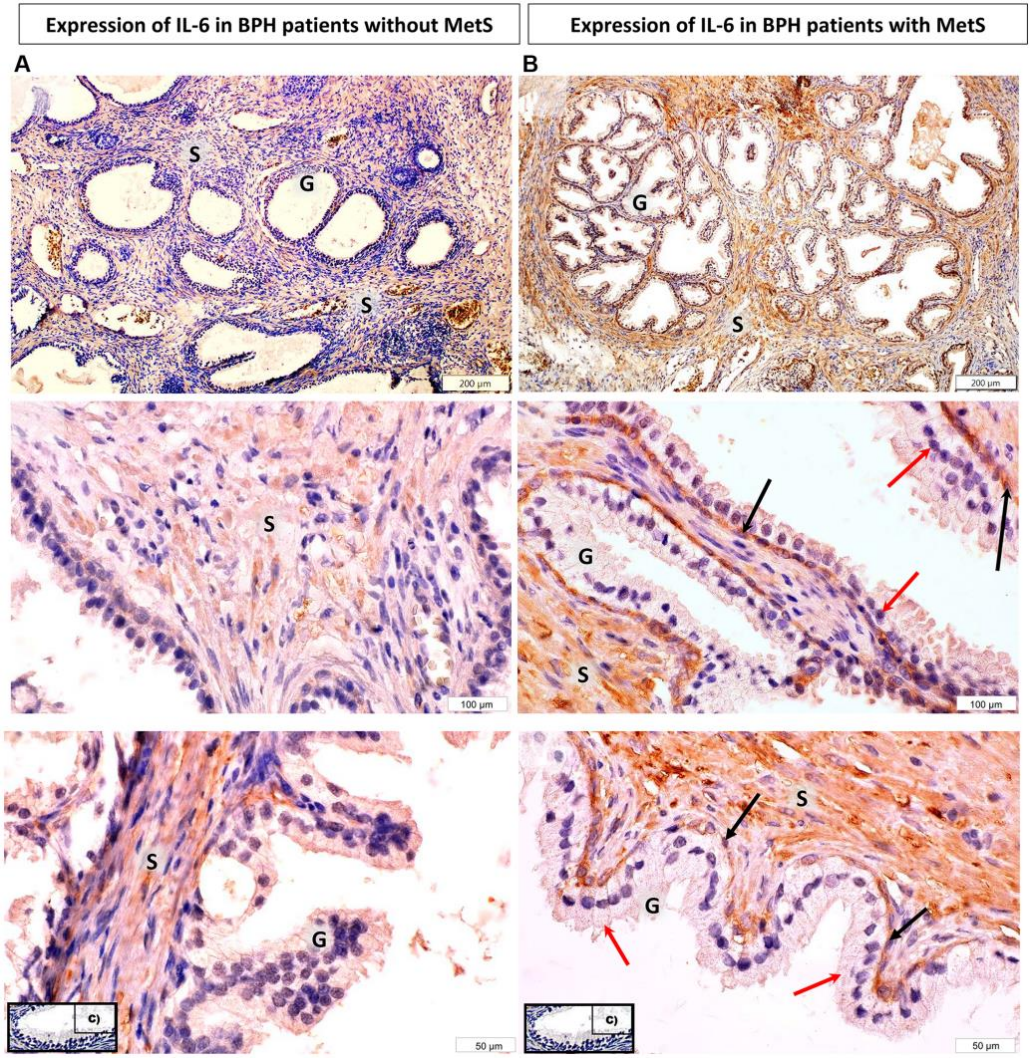
**Microscopic image of the expression of the cytoplasmic immunohistochemical reaction to IL-6.** (**A**) microscopic specimen of the prostate in a patient with benign prostatic hyperplasia and without MetS, (**B**) microscopic specimen of the prostate in a patient with benign prostatic hyperplasia and MetS. C - negative control (reaction without the use of an antibody). The cytoplasmic immunohistochemical reaction expressing IL-6 was stained brown (DAB +), S - prostate stroma, G - glandular part, ↑ - luminal cells, ↑ basal cells.

**Figure 2:**
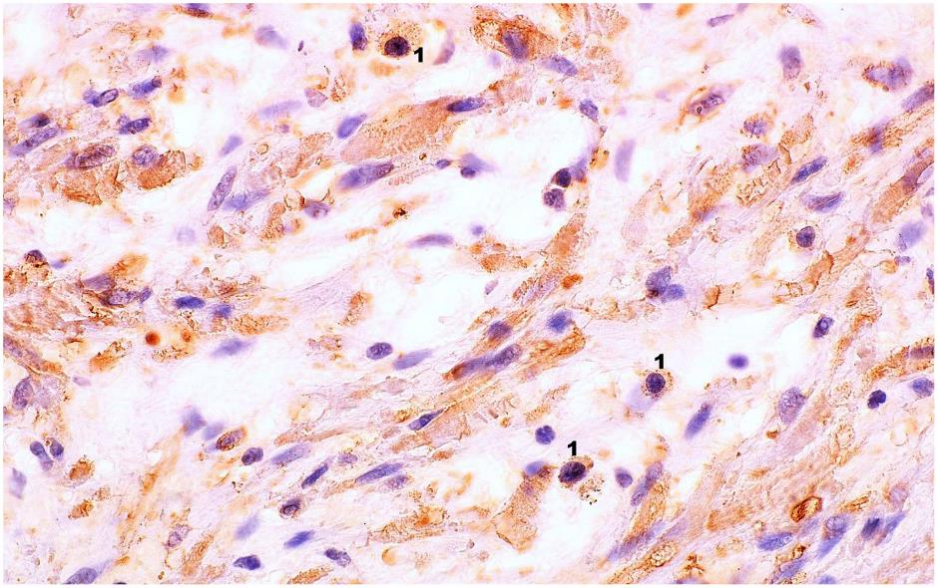
**Microscopic image of the expression of the cytoplasmic immunohistochemical reaction to IL-6.** Microscopic preparation of prostate stroma tissue with benign hyperplasia. Cytoplasmic immunohistochemistry showing IL-6 expression was stained brown (DAB +), 1 – lymphocytes, 600× magnification.

**Figure 3:**
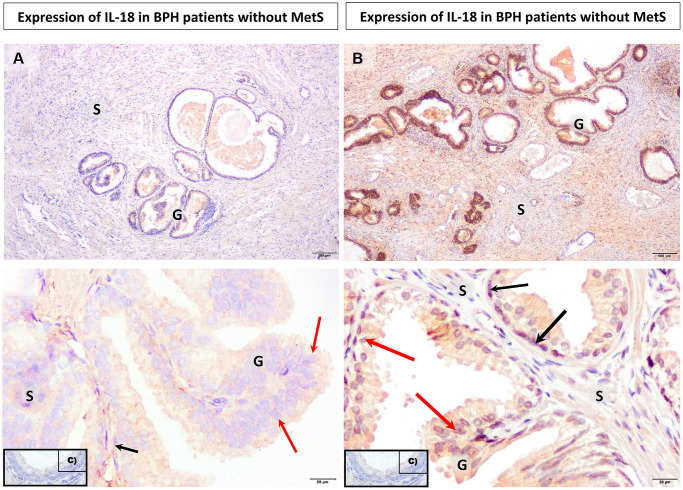
**Microscopic image of the expression of the cytoplasmic immunohistochemical reaction to IL-18.** (**A**) microscopic specimen of the prostate in a patient with benign prostatic hyperplasia and without MetS, (**B**) microscopic specimen of the prostate in a patient with benign prostatic hyperplasia and MetS, C - negative control (reaction without the use of an antibody). The cytoplasmic immunohistochemical reaction expressing IL-18 was stained brown (DAB +), S - prostate stroma, G - glandular part, ↑ luminal cells, ↑ basal cells.

The immunoexpression of IL-6 and IL-18 in the prostate tissue obtained from patients with clinically and histopathologically confirmed BPH was visible in the form of the brown-stained cytoplasm of basal cells and luminal cells of the prostate epithelium, and in the prostate stroma (Figures 1–3).

#### Increased IL-6 immunoexpression in the cytoplasm of prostate stromal cells with benign hyperplasia

**Figure 4:**
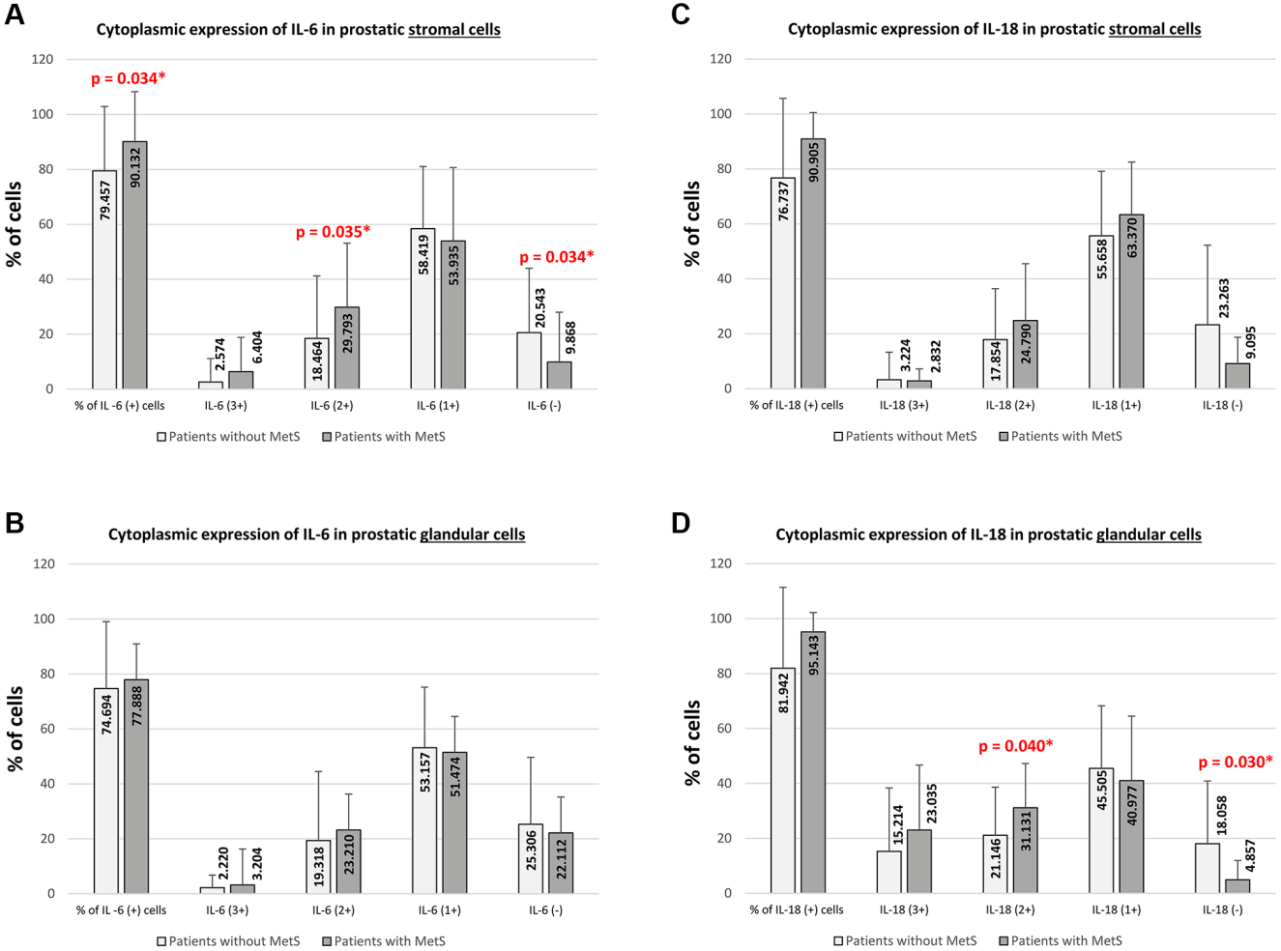
**Increase in cytoplasmic immunoexpression of IL-6 and IL-18 in prostate tissue with benign hyperplasia in MetS patients.** (**A**) IL-6 expression levels significantly increased in prostate stromal cells of BPH + MetS patients. (**B**) No significant changes in IL-6 immunoexpression were observed in prostate epithelial cells, regardless of group. (**C**) No significant changes in IL-18 immunoexpression were also observed in the prostate stroma (**D**), while in the case of glandular epithelial cells, an increase in the percentage of IL-18 positive cells was observed in the BPH + MetS group.

In the prostate stroma, depending on the presence of the metabolic syndrome, differences between the studied groups were observed. In prostatic tissue cells from patients BPH + MetS, the mean percentage of IL-6 positive (+) cells was statistically significantly higher than in patients BPH (*p* = 0.034). Moreover, in the BPH + MetS group, the mean percentage of cells showing strong IL-6 expression (2+) was statistically significantly higher than in the group without MetS (*p* = 0.035) (Supplementary Table 1, Figure 4A).

Unlike the stromal cells of the prostate, in prostate glandular epithelial cells, no statistically significant differences were found in the overall percentage of IL-6 (+) cells and in any of the levels of intensity of immunohistochemical expression between patients without MetS and with MetS (Supplementary Table 1, Figure 4B).

The obtained results indicate that IL-6 immunoexpression is much stronger in the cytoplasm of prostate stromal cells in BPH + MetS patients. Which may indicate the participation of this interleukin in proliferative processes in the stroma of the prostate in patients with metabolic disorders.

#### Increased IL-18 immunoexpression in prostate glandular epithelial cells with benign hyperplasia

In prostate stromal cells, no statistically significant differences were found in the overall percentage of IL-18 (+) cells, IL-18 (−) cells, and in any of the levels of intensity of immunohistochemical expression between the patients without MetS and with MetS (Supplementary Table 2, Figure 4C).

Unlike the stromal cells of the prostate, in prostate glandular epithelium cells, depending on the presence of MetS, differences between the studied groups were observed. In prostatic tissue of patients from BPH + MetS group, the percentage of cells showing strong IL-18 expression (2+) was statistically significantly higher than in the group of BPH patients (*p* = 0.040). It was also shown that in the BPH group of patients, the mean percentage of cells not showing IL-18 expression (−) was significantly higher than in the BPH + MetS group (*p* = 0.030). In prostate glandular epithelium cells, no difference was shown in the total percentage of IL-18 (+) cells, the percentage of cells showing very strong IL-18 expression (3+) and weak IL-18 expression (1+) (Supplementary Table 2, Figure 4D). Our results indicate that IL-18 immunoexpression is stronger in the cytoplasm of prostatic glandular epithelial cells in the BPH + MetS group. This indicates that in patients with metabolic disorders, IL-18 plays a more significant role in the proliferation of the glandular epithelium than IL-6.

### Immunoexpression of IL-6 and IL-18 in benign prostatic cells is associated with TG and HDL

#### Interleukin-6

The correlation analysis showed that the level of IL-6 immunoexpression in the patients with BPH, both without and with MetS, correlated only with one of lipid parameters analysis, HDL concentrations (Supplementary Table 3).

In the BPH group, in the prostate stroma, HDL levels correlated negatively with the overall percentage of IL-6 (+) cells (R = −0.312, *p* = 0.050) and positively with the percentage of IL-6 (−) cells (R = 0.312, *p* = 0.050). Moreover, in the prostate glandular epithelium, HDL levels correlated negatively with the overall percentage of IL-6 (+) cells (R = −0.400, *p* = 0.019), with cells showing moderate IL-6 (2+) expression (R = −0.404, *p* = 0.018) and positively with the percentage of cells that did not show IL-6 expression (R = 0.400, *p* = 0.019) (Supplementary Table 3).

In the patients with BPH + MetS, the correlation was observed only in stroma cells, for HDL levels and the percentage of IL6 (3+) cells (R = 0.341, *p* = 0.049) and IL-6 (2+) (R = 0.379, *p* = 0.027) (Supplementary Table 3).

Therefore, it seems that in patients with BPH + MetS, HDL as an isolated lipid indicator loses its importance as a protective factor against inflammation in the body.

#### Interleukin-18

The correlation analysis showed that the level of IL-18 immunoexpression in BPH patients, depending on the presence of MetS, correlated with the concentrations of TG and HDL (Supplementary Table 4).

In the patients with BPH, in the prostate stroma, the level of TG correlated positively with the overall percentage of IL-18 (+) cells (R = 0.396, *p* = 0.007), the percentage of IL-18 (3+) cells (R = 0.496, *p* = 0.001), the percentage of IL-18 (2+) cells (R = 0.436, *p* = 0.003), and negatively with IL-18 (−) cells (R = −0.400, *p* = 0.006). Additionally, HDL levels were also shown to correlate negatively with the overall percentage of IL-18 (+) cells (R = −0.362, *p* = 0.015) and the percentage of IL-18 (2+) cells (R = −0.377, *p* = 0.011) and positively with the percentage of IL-18 (−) cells (R = 0.357, *p* = 0.016). However, in the prostate glandular epithelium, only HDL level correlated negatively with the overall percentage of IL-18 (+) cells (R = −0.376, *p* = 0.034) and positively with the cells showing low IL-18 expression (R = 0.414, *p* = 0.018).

In the patients with BPH + MetS, relationships were observed only with respect to prostate glandular epithelial cells. The TG level negatively correlated only with IL-18 (1+) cells (R = −0.505, *p* = 0.014). On the other hand, HDL levels correlated negatively with the percentage of IL-18 (3+) cells (R = −0.454, *p* = 0.029) and positively with the percentage of IL18 (1+) cells (R = 0.512, *p* = 0.013). Only in this case, a negative correlation of glucose concentration with the percentage of IL-18 (3+) cells was observed (R = −0.416, *p* = 0.049) (Supplementary Table 4).

In the case of IL-18 expression, the interaction of TG and HDL is much more noticeable in the BPH group without MetS. An increase in the concentration of TG is noticeable with an increase in the percentage of cells with cytoplasmic expression of IL-18 in the prostate stroma. However, in the case of HDL, lower HDL concentrations are associated with an increase in the percentage of cells expressing IL-18 in this tissue.

### IL-18 immunoexpression in patients with BPH + MetS is associated with TG/HDL and LDL/HDL ratio

In the prostate stromal and glandular epithelial cells, regardless of the presence of MetS, no statistically significant relationships were found between the overall percentage of IL-6 (+) cells, IL-6 (−) cells and the levels of intensity of immunohistochemical expression, and the TG/HDL and LDL/HDL ratio (Supplementary Table 5).

However, in the case of IL-18, the correlation analysis showed that in the patients with BPH, in prostate epithelial cells, the percentage of IL-18 (1+) cells negatively correlated with both the TG/HDL ratio (R = −0.500, *p* = 0.015) and the LDL/HDL ratio (R = −0.375, *p* = 0.034) (Supplementary Table 6).

Much more relationships were observed in prostatic tissue collected from the patients with BPH + MetS. In the case of stromal cells, positive correlations were found between the TG/HDL ratio and the overall percentage of IL-18 (+) cells (R = 0.439, *p* = 0.003), the percentage of IL-18 cells (3+) (R = 0.466, *p* = 0.001), the percentage of IL-18 (2+) cells (R = 0.459, *p* = 0.002) and a negative correlation with the percentage of IL-18 (−) cells (R = −0.442, *p* = 0.002), while in the prostate glandular epithelium there were positive relationships between the TG/HDL ratio and the overall percentage of IL-18 (+) cells (R = 0.399, *p* = 0.024) and the percentage of IL-18 (2+) cells (R = 0.366, *p* = 0.039). Moreover, a positive relationship was demonstrated between the LDL/HDL ratio and the percentage of IL-18 (3+) cells (R = 0.433, *p* = 0.039) (Supplementary Table 6).

These data indicate that not only TG and HDL concentrations are related to IL-18 immunoexpression in benign prostatic tissue. In patients with BPH + MetS, the TG/HDL and LDL/HDL ratios are crucial for the development of the IL-18-mediated inflammatory state. The obtained results also show that in patients with BPH + MetS, lipid parameters are most involved in the development of local inflammation. There is also an observation, requiring further research, that lipid indices show a stronger relationship with the expression of selected pro-inflammatory indices in the prostate tissue with hyperplasia (and thus accompanying a local inflammatory process) in patients with BPH and generalized inflammation resulting from the coexistence of MetS.

### Increased expression of IL-6 in prostate tissue with benign hyperplasia is associated with SCFAs - C2:0, C4:0n, C6:0n

The correlation analysis showed a relationship between the amount and intensity of IL-6 expression in benign prostatic hyperplasia tissue cells and the percentage of SCFAs isolated from the stool of patients (Supplementary Table 7).

In the group of BPH patients, it was shown that only in glandular epithelial cells, the overall percentage of IL-6 (+) cells correlated weakly, negatively with caproic acid (C6:0n) (R = −0.397, *p* = 0.020).

In turn, in the group BPH + MetS, correlations were found in both the stromal cells and prostate glandular epithelial cells. In the stromal cells there was a weak positive correlation between IL-6 (1+) cells and acetic acid (C2:0) (R = 0.381, *p* = 0.026) and a weak negative correlation between IL-6 (1+) cells and butyric acid (C4:0n) (R = −0.377, *p* = 0.028). On the other hand, in the cells of the prostate glandular epithelium there was a moderate positive correlation between IL-6 (1+) cells and acetic acid (C2:0) (R = 0.504, *p* = 0.007) and a moderate negative correlation between IL-6 (1+) cells and butyric acid (C4:0) (R = −0.643, *p* < 0.001) (Supplementary Table 7).

In the BPH + MetS group, among the SCFAs that are associated with IL-6 expression in prostate tissue, an increase in C2:0 results in an increase in the percentage of IL-6 (+) cells. Whereas, C4:0 shows a negative correlation with IL-6 (+) cells. This indicates that disturbances in the concentration of butyric acid, which is crucial for the intestinal microenvironment, may be associated with the production of benign inflammation in the prostate gland in BPH + MetS patients.

### Increased expression of IL-18 in prostate tissue with benign hyperplasia is associated with SCFAs - C2:0, C6:0i

The correlation analysis showed a relationship between the amount and intensity of the IL-18 expression in the benign prostatic hyperplasia tissue and the percentage of SCFAs isolated from the stool of patients (Supplementary Table 8).

In the group of BPH patients, it was shown that only in glandular epithelial cells, the number of cells with a strong (3+) cytoplasmic reaction to IL-18 correlates moderately positively with isocaproic acid (C6:0i) (R = 0.421, *p* = 0.021).

In the BPH + MetS group, a relationship was also demonstrated only between prostate glandular epithelial cells and SCFAs. The overall percentage of IL-18 positive (+) cells correlated moderately negatively with acetic acid (C2:0) (R = −0.424, *p* = 0.044) (Supplementary Table 8).

In patients with BPH, a significant positive relationship was found between C6:0i (inflammatory in nature) and very strong expression of IL-18 in the glandular epithelium. Which proves that C6:0i affects the production of an inflammatory microenvironment in the glandular part of the prostate dependent on IL-18. On the other hand, the negative relationship between C2:0 and the percentage of cells expressing IL-18 in the BPH + MetS group indicates that it may play an anti-inflammatory and protective role before the formation of inflammation.

## DISCUSSION

There is a large body of literature on prostate inflammation as a common denominator for the development of prostate diseases. The results of epidemiological and histopathological studies and laboratory tests also indicate that inflammation is an extremely crucial element in the pathogenesis and progression of benign prostatic hyperplasia (BPH) and prostate cancer (PCa). It turns out that chronic and low-level inflammation of the gland is involved in the pathogenesis and progression of both disease entities. These studies also indicate that inflammation of the prostate gland should be a special and new field of basic and clinical research in patients with BPH and PCa [10]. Chronic inflammation resulting from exposure to pathogenic and environmental factors is the cause of neoplastic diseases, including such diseases of stomach, liver or colon. In the prostate gland, chronic inflammation is initiated by many stimuli – both well-known (including bacterial and viral sexually transmitted infections) and unknown factors that influence the onset of inflammation, which have a pro-inflammatory effect in the prostate microenvironment. Prostatitis may also be a result and part of the systemic inflammation caused by obesity and the metabolic syndrome in men. The metabolic syndrome and its components contribute to the persistence of chronic inflammation at a low level, and thus are associated with the presence of systemic markers of inflammation (including leptin, IL-6, TNF), which may contribute to the development of proliferative and inflammatory processes. Neoplastic tumours in the prostate [28]. There are also histological studies in which chronic or acute inflammation has been demonstrated in 100% of BPH tissue samples tested. In addition, it was also found that inflammatory infiltrates in the prostate tissue with benign hyperplasia are associated, among others, with a significant increase in the prostate volume, more severe symptoms from the lower urinary tract, sudden urinary retention and proliferation of epithelial cells [29].

Interleukin 6 is a pro-inflammatory cytokine responsible mainly for the activation of B lymphocytes. Its production occurs mainly in areas of sudden and chronic inflammation. It has been shown that IL-6 is expressed in both stromal cells and epithelial cells of the prostate tissue with benign hyperplasia. Studies have also confirmed that IL-6 increases significantly in prostate cancer tissue, where it plays the role of a growth factor for cells [30, 31]. The studies by Penn et al. [32] showed that the stromal cells of the human prostate with benign hyperplasia can activate specific CD4 + T cells for the production of IFNγ and IL-17, which in turn induces the secretion of IL-6 and IL-8 that are key growth factors for epithelial cells and prostate stromal cells. The consequence of such action on prostate cells will be their hyperproliferation and promotion of prostate hyperplasia [32]. In a study by Hobisch et al. [31] carried out with the use of an immunohistochemical method in the prostate tissues with benign hyperplasia, the expression of IL-6 in the cytoplasm of epithelial cells was demonstrated. In addition, it was also found that IL-6 expression predominated in basal cells. However, no positive reaction was found in the stroma of the tissue, while a positive reaction was observed in atrophic areas [31]. In our study, the tissue expression of IL-6 was also confirmed in the prostate tissue, both in part of the glandular epithelium and in the area of prostate stromal cells. Additionally, the differences in IL-6 pro-inflammatory expression were analysed in patients depending on the occurrence of metabolic syndrome. The results of this analysis indicate that in the tissue of patients with BPH and MetS, the overall percentage of cells showing IL-6 expression in prostate stromal cells is significantly higher than in patients without MetS. So far, no studies have been conducted to analyse the effect of MetS on the level of IL-6 expression in the prostate tissue with benign hyperplasia. The relationship between the concentrations of SCFAs isolated from patients’ faeces and the tissue expression of pro-inflammatory interleukins (IL-6 and IL-18) was not analysed either. The studies conducted so far, where the expression of IL-6 was confirmed in the immunohistochemical reaction, mainly concerned the differences between BPH and prostate cancer [33]. These studies have shown that in the case of benign prostatic hyperplasia, high tissue IL-6 expression depends on a high concentration of secreted PSA [33]. The studies comparing the factors proving the presence of inflammation analysed, among others, IL-6 concentration [34]. The results of these studies clearly show that both in patients with BPH and with prostate cancer, compared to men in the control group, there are statistically significant higher concentrations of pro-inflammatory IL-6 [34]. These studies confirm that IL-6 is involved in the initiation of BPH and influences its course. Moreover, when measured in serum, it may be a potential factor that differentiates inflammatory prostate hyperplasia from healthy prostate tissue [34]. Other studies that also analysed biomarkers of general inflammation, including IL-6, indicate that increased concentrations of this parameter in the blood serum of patients are associated with an increased risk of developing BPH and more severe symptoms during its progression [29]. The same studies also indicated that obesity in patients with BPH is a factor contributing to the occurrence of systemic inflammation, which is reflected in the increased concentration of pro-inflammatory factors [29].

In our study, no statistically significant difference was found between the mean concentrations of IL-6 in the blood serum of patients, even though the mean values of the studied parameter were higher in patients with BPH than in men from the control group. Also, no inter-patient differences were observed when IL-6 levels were compared between groups depending on the coexistence of MetS.

The metabolic syndrome is associated with the occurrence of chronic inflammation that remains at a low level, which is characterised by the predominance of pro-inflammatory factors in the systemic circulation, e.g., IL-6 and TNFα [35–37] and IL-18 [22, 38], which are involved in the pathogenesis of LUTS/BPH [39, 40]. Another factor that may also affect the onset of systemic inflammation in the body and, consequently, the onset and progression of BPH, is obesity and the abdominal adipose tissue (MetS component), secreting adipokines [41]. The presence of MetS in patients with BPH is significantly associated with an increase in the prostate volume and its antero-posterior dimension, which significantly reduces the urethral flow assessed in uroflowmetry [5]. However, in our study, although the levels of serum interleukins – IL-6 and IL-18, were higher in patients with BPH and with MetS, these differences were not statistically significant.

In a study by Gacci et al. [5] it was also confirmed that the occurrence of the metabolic syndrome is associated with an increase in the prostate volume and inflammation in the prostate tissue with benign hyperplasia. Moreover, among the diagnostic factors for MetS, increased serum TG levels and decreased HDL levels (dyslipidaemia) were significantly associated with an increased risk of developing an enlarged gland (PV> 60 cm^3^). Another study by Gacci et al. [2] also confirm that the main factor increasing the risk of prostate enlargement is too low HDL level. Studies conducted in the Chinese population also confirm that diagnostic factors for MetS, the presence of abdominal obesity and low HDL levels are the main factors significantly associated with the occurrence of BPH [42]. Another multivariate analysis showed that, regardless of age, MetS was associated with a higher incidence of BPH. It has also been shown again that HDL is the MetS component that shows the strongest relationship with the spread of BPH in men [43]. Similar results were obtained in a study by Yoo et al. [44], in which among men not taking statins, it was shown that HDL liporptotein levels higher than 60 mg/dL reduce the incidence of BPH, while HDL concentrations below 40 mg/dL, especially in men under 40, predispose to BPH. Moreover, decreased HDL concentrations are significantly associated with the severity of LUTS symptoms [45]. In our study, it was found that HDL levels significantly correlate with the percentage of IL-6 cells showing immunoexpression in BPH tissue cells, which confirms the results of previous studies and the relationship of MetS inflammation to the onset and development of BPH. Additionally, an incorrect TG/HDL ratio also correlates with an increase in the prostate volume [46]. In our study, we did not show a relationship between the TG/HDL and LDL/HDL ratios and the immunoexpression of IL-6 in the prostate tissue, regardless of the presence of MetS in patients. However, such relationships were observed in the case of IL-18 immunoexpression in stromal and prostate epithelial cells, mainly in patients with BPH and MetS. The results of our study indicate that low HDL levels and an increased TG/HDL ratio are present in patients with BPH and MetS, and additionally contribute to the formation of an inflammatory microenvironment, as evidenced by the tissue immunoexpression of IL-18. Moreover, too low levels of anti-inflammatory HDL may be involved in the activation of the inflammasome [47], resulting in the expression of just IL-18 [48].

Interleukin 18 is produced mainly by macrophages and may mediate an anti-tumour immune response, and also induces the secretion of gamma interferon [49]. In molecular study, Hamakawa et al. [20] also showed the expression of IL-18 in stromal and epithelial cells of the prostate with BPH. In addition, the receptor for IL-18 was also found to be localised in prostate smooth muscle cells. Additionally, it was found that IL-18 is directly involved in the pathogenesis of BPH by inducing thrombospondin-1 (TSP-1), which promotes the proliferation of human prostate stromal cells [20]. The study by Steiner et al. [50] confirmed that the pro-inflammatory cytokines IL-2 and IFNγ as well as IL-15 [51], IL-17 [13] are significantly increased in the benign prostatic hyperplasia tissue, which also proves the presence of IL-18. The role of interleukin 18 and its pro-inflammatory properties are also analysed in relation to prostate cancer and its various stages of advancement, including its metastatic potential. So far it has been confirmed that IL-18 determined in the blood serum of patients can be a very helpful marker for monitoring the stage of prostate neoplastic disease. The studies show that the concentration of IL-18 in the blood serum of patients with prostate cancer is significantly higher than in healthy patients from the control group (345 ± 94.8 pg/ml vs. 180 ± 54.1 pg/ml, *p* < 0.05) [52].

Short-chain fatty acids work in the body by activating four different receptors located in the membranes of cells. It is well known that SCFAs and their receptors modulate inflammation in the gut. Among them, there are receptors belonging to the GPCR family: GPR43 receptor (FFAR2, free fatty acid receptor 2), GPR41 receptor (FFAR3, free fatty acid receptor 3), GPR109a receptor (HCA2, hydroxycarboxylic acid receptor 2) as well as the Olfr-87 receptor [53–55]. The GPR43 receptor is mainly stimulated by acetic and propionic acid, but also by butyric, valeric and formic acid. On the other hand, propionic, butyric, valeric and caproic acids have the greatest potential to activate GPR41, while acetic and formic acids are slightly less effective. The ligand for the GPR109a receptor, apart from niacin and ketone bodies, is butyric acid and β-hydroxybutyric acid [56]. The main site of GPR43 expression is epithelial cells of the gastrointestinal tract, cells of the immune system and adipocytes [57, 58]. In turn, the GPR41 receptor is present on many cells of the human body, including lamina propria cells of the colon, spleen cells, lymph nodes, bone marrow, adipocytes, PMN cells or in cells of the peripheral nervous system [57]. The presence of GPR109a receptor has been confirmed in the colon epithelium, but also in immunocompetent cells – macrophages, monocytes, dendritic cells (DC), neutrophils and adipocytes [57].

With regard to the prostate, the most important receptor is Olfr-78, known in humans as OR51E2 (olfactory receptor 51E2) [59, 60], which is activated by acetic and propionic acid. It has been suggested that this receptor in the prostate tissue may influence the chronic inflammatory response leading to intraepithelial neoplasia and prostate cancer [61]. Moreover, it is also present in renal vessels involved in renin secretion [62], on neurones and enteroendocrine cells [63].

In our previous study, patients with BPH had significantly lower mean concentrations of isocaproic acid (C6:0i) compared to patients in the control group (0.359 vs. 0.186, *p* = 0.038) (data shown in [27]), which is consistent with the results obtained in patients with IBD with lower concentrations of C6 acid [64]. In our study, the relationship between the analysed tissue reactions of pro-inflammatory interleukins and short-chain fatty acids was demonstrated for the first time. A negative weak relationship was demonstrated between the total percentage of IL-6 (+) cells and caproic acid (C6:0n) in patients without MetS, and between butyric acid (C4:0n) and IL-6 (1+) cells in both stromal cells and the glandular epithelium of the prostate with benign hyperplasia in patients with MetS. The study also shows that acetic acid (C2:0) positively correlates with IL-6 (1+) cells in both stromal cells and prostate glandular epithelium with benign hyperplasia in patients with MetS. This seems to be important due to the presence of the ORF51E2 receptor in prostate cells, which is stimulated by acetic acid. Acetate, like propionate and butyrate, has a proven anti-inflammatory effect. In the study by Tedelind et al. [65] it was confirmed that this acid has the properties of inhibiting the secretion of TNFα and IL-6 and reduces the activity of NFκB from tissue culture, and thus may inhibit the ongoing inflammatory process. The results of this study may indicate that in the data obtained by us, acetic acid also has a similar effect in the prostate tissue collected from patients with BPH and with MetS. In this group of patients, a high percentage of cells showing the overall percentage of IL-6 (+) cells, which was influenced by the percentage of IL-6 (1+) cells, was observed in the stromal and glandular epithelial cells. In turn, IL-6 (3+) cells were observed the least.

Our study also found a negative correlation between the overall percentage of IL-18 (+) cells in the glandular epithelium of benign prostatic hyperplasia tissue in the group of patients with MetS and the amount of acetic acid in the faeces. So far, no studies have analysed this relationship. It is known, however, that acetate has an anti-inflammatory effect and may, among others, reduce the activation of the NLRP3 inflammasome, which is involved in the production of IL-18, and thus reduce its amount [66]. The role of the inflammasome and the secretion of IL-18 with it was first confirmed in studies conducted in rats. It has been shown that prostate inflammation in the course of BPH is associated with the activation of the inflammasome, and through the secretion of pro-inflammatory interleukins, it is involved in maintaining the inflammation of the prostate gland [19].

Referring this to the results obtained by us, it can be stated that in the case of the prostate tissue collected from BPH patients with MetS, the negative relationship between acetate and IL-18 does not reduce its cellular synthesis, which is observed as the highest percentage of IL-18 positive cells in the glandular epithelium of the prostate. The tissue expression of IL-18 was also confirmed in the study by He et al. [67], in which its relationship with low values of omentin-1 – a hormone synthesised by adipose tissue, the reduced concentrations of which were observed, among others, was analysed in type 2 diabetes, obesity or cardiovascular diseases. It has also been proven that IL-18 significantly contributes to the development of inflammatory diseases or chronic inflammation in the course of BPH [67]. The study by Lebel-Binay et al. [68] analysed the secretion of IL-18 in the prostate tissue. It was confirmed that in healthy prostate, IL-18 is expressed in basal cells and to a very small extent in stromal cells, while epithelial cells did not show IL-18 expression. Similar results were obtained in the examined prostate tissue with benign hyperplasia, where the expression of IL-18 was visible only in the basal cells of the prostate glandular epithelium. On the other hand, in the tissue with prostate cancer, a positive IL-18 inflammation was found in the cytoplasm of neoplastic cells covering the prostate glandular epithelium and in the stromal cells of the prostate [68]. The concentration of IL-18 in the serum of patients participating in the study was comparable to the concentrations obtained in healthy subjects. The increase in IL-18 expression was noticeable only in the prostate tissue (glandular epithelium) with benign hyperplasia in the BPH + MetS group, what indicates the development of local inflammation.

## CONCLUSIONS

The results of the conducted research indicate that the MetS components, in particular the HDL and TG levels, are associated with the occurrence of BPH in ageing men. Additionally, these parameters influence the formation of inflammation in the prostate tissue, which is one of the factors activating the signalling pathways that stimulate the cells of the stromal and glandular epithelium to proliferate. It has also been demonstrated for the first time that, indirectly, through SCFAs, the gut microbiota can act to prevent or create an inflammatory microenvironment in the prostate gland. Therefore, it seems important to prevent local inflammation in patients, i.a., by inhibiting IL-6 or IL-18. In addition, in patients with BPH and aging men who may develop prostate diseases, attention should be paid to the state of the composition and functioning of the intestinal microbiota, which may directly, but also indirectly, affect the immunological processes taking place in the prostate gland. Intestinal microbiota affects the entire immune system, and thus individual tissues and organs. Creating a properly functioning intestinal microenvironment can significantly reduce the overall inflammation, which also affects the prostate. However, in order to fully elucidate the mechanism of interaction between lipid metabolism disorders, short-chain fatty acids, and the formation of an inflammatory microenvironment in the prostate tissue, further *in vivo* and *in vitro* studies are needed.

## MATERIALS AND METHODS

### Study groups and inclusion/exclusion criteria

#### Patients

The study involved 103 men diagnosed with and treated for BPH, who had undergone transurethral resection of the prostate (TURP) at the Clinic of Urology and Urological Oncology, Pomeranian Medical University, Szczecin, Poland between November 2017 and May 2019. The men were aged 49–79 years (mean age: 66.4). The diagnosis was based on the results of the International Prostate Symptom Score (IPSS) questionnaire (with one question relating to overall quality of life - QoL), long lasting symptoms, decreased flow rate (Q_max_) or urinary retention, and increased prostate volume. BPH was confirmed in the prostate tissue removed from the prostate gland during the TURP procedure.

**Figure 5:**
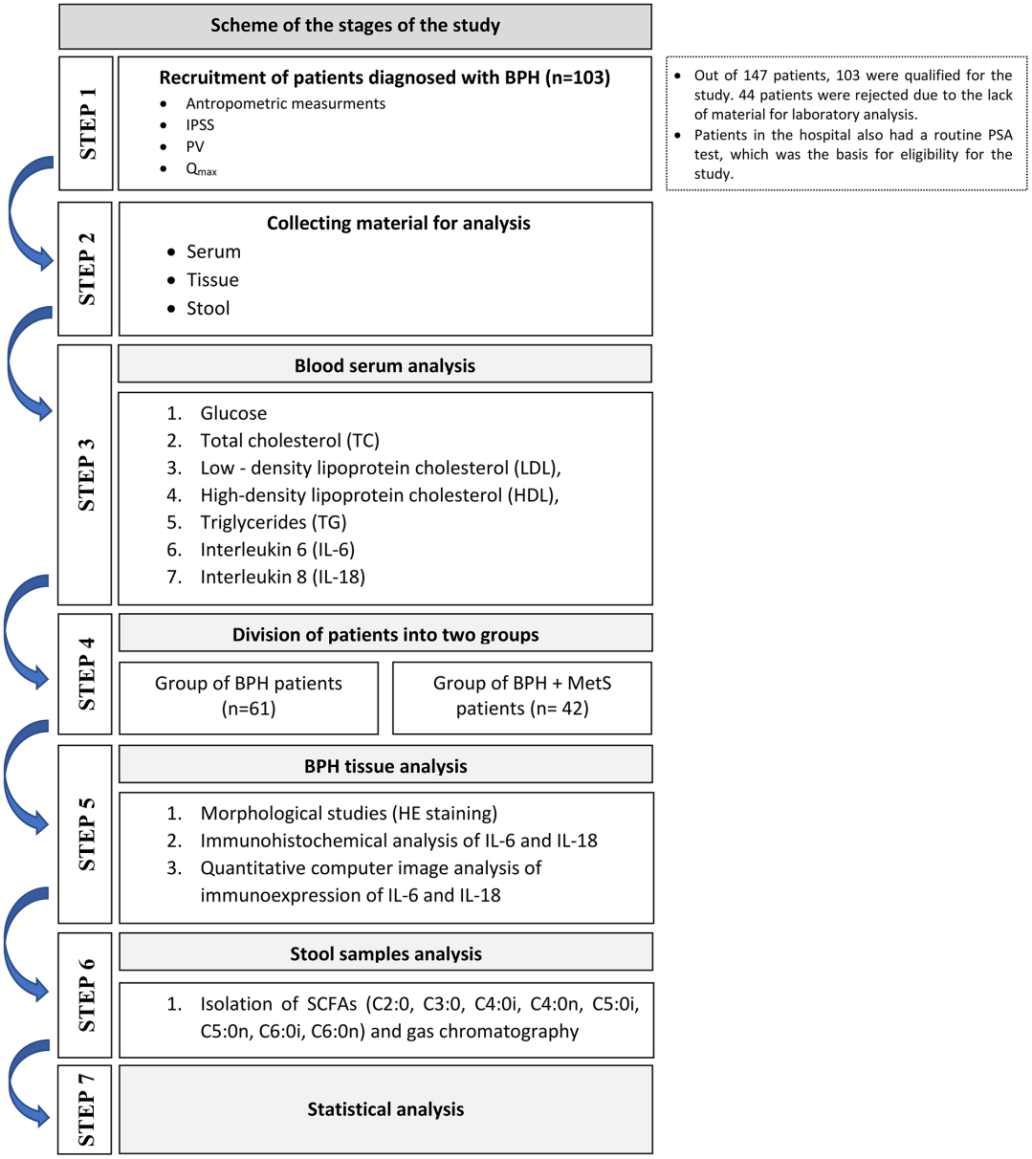
Study flow chart.

The criteria for exclusion from the study were: active cancer disease, alcoholism, thyroid diseases, taking glucocorticosteroids and antibiotics for six months preceding the examination. Only those patients from whom we obtained a complete set of material for laboratory tests (serum and stool sample) were included in the research. The flowchart of the entire experiment is presented below (Figure 5).

### Clinical examination

Anthropometric measurements were performed in all patients – body weight, height and waist circumference. The participants of the study completed questionnaires concerning demographic data and health status. Based on the criteria presented by the International Diabetes Federation (IDF) in 2005 [69], the men were divided according to the presence of MetS. The men with abdominal obesity were qualified for the MetS group if they had waist circumference ≥ 94 cm and at least two of the following abnormalities: triglycerides (TG) ≥ 150 mg/dl or treatment for *dyslipidaemia*; high-density lipoprotein (HDL) cholesterol < 40 mg/dl or treatment for dyslipidaemia; fasting glycaemia ≥ 100 mg/dl or treatment for type 2 diabetes; *blood pressure* ≥ 130/85 mmHg or treatment for hypertension. All components of MetS were considered both individually and as sets of symptoms.

### Blood serum analysis

#### Biochemical parameters and interleukin-6 and -18

To evaluate basic biochemical parameters, such as the serum levels of glucose, total cholesterol, low-density lipoprotein cholesterol (LDL), high-density lipoprotein cholesterol (HDL), triglycerides (TG) and interleukin-6 and interleukin-18. We collected blood using the Vacutainer system tubes with clot activator and gel separator. 7.5 ml blood samples were taken from a cubital vein on an empty stomach between 7:30 am and 9:00 am. The blood was collected by qualified medical staff and delivered to the laboratory in accordance with the relevant rules and procedures. The parameters were determined using a spectrophotometric method (biochemical parameters) and enzyme-linked immunosorbent assay (ELISA) (interleukins) with commercial reagent kits, in accordance with the protocols attached thereto.

### Morphological studies

The prostate sections were routinely fixed in 4% buffered paraformaldehyde, and embedded in paraffin. The paraffin blocks were cut into 4 μm thin sections, which were next stained using a standard haematoxylin and eosin method (HE; Sigma-Aldrich, Germany). The samples were examined under an Olympus BX46 light microscope (Olympus Optical Co., Ltd., Tokio, Japan).

### Immunohistochemical analysis

BPH sections were deparaffinised, rehydrated. In order to retrieve antigens, the slides were boiled for 30 minutes in Target Retrieval Solution Citrate at pH 6.0 (Target Retrieval Solution, Citrate pH 6, Dako, Glostrup, Denmark, cat. No. S2369) for IL6 and at pH 9.0 (Target Retrieval Solution, pH 9 (10X), Dako, Glostrup, Denmark, cat. No. S2367) for IL-18. Following this, they were washed in PBS. The activity of endogenous peroxidase was blocked by using peroxidase blocking solution (Dako, Glostrup, Denmark) for 10 minutes at room temperature. Sections were then incubated overnight at 4°C in a humid chamber with a mouse IgG anti-IL-6 monoclonal antibody (Biorbyt Ltd., Cambridge, United Kingdom, cat. No. orb303674) at a concentration of 0.150 μg/ml. For IL-18, sections were incubated for 30 minutes at room temperature in a humid chamber with a mouse polyclonal IgG antibody against IL-18 (Biorbyt Ltd., Cambridge, United Kingdom, cat. No. orb129642) at a concentration of 1:100 (10 μg/ml). Primary antibodies were diluted in antibody diluter (Dako, Glostrup, Denmark, cat. No. S0809). Next, the sections were incubated with a complex containing secondary antibody conjugated with horseradish peroxidase (Dako, Glostrup, Denmark, EnVision Detection Systems, Peroxidase/DAB, Rabbit/Mouse, cat. No. K5007). After washing out the secondary antibody, diaminobenzidine was applied for 5–10 minutes (1:50 ratio, Dako, Glostrup, Denmark). As the final step, the slides were counterstained with Mayer’s haematoxylin (Sigma-Aldrich Co., St. Louis, MO, USA), dehydrated, and cover-slipped. Washing the slides with phosphate-buffered saline preceded each step of the procedure. The slides were examined under a light microscope (Olympus BX 41, Hamburg, Germany). The negative controls for reaction specificity were performed.

### Quantitative computer image analysis of immunoexpression of IL-6 and IL-18

Using the ScanScope AT2 scanner (Leica Microsystems, Wetzlar, Germany) IL-6 and IL-18 immunostained BPH sections were scanned at a magnification of 400× (resolution of 0.25 μm/pixel). The obtained digital images of the myomas were analysed on a computer screen using ImageScope viewer software (Aperio Technologies, Vista, CA, USA).

The quantitative analysis of IL-6 and IL-18 immunoexpression was performed on slides using the cytoplasmic v9 and positive pixel count algorithm (Aperio Technologies, Vista, CA, USA). Other parameters were set to achieve compliance with the visual assessment of colour intensity. The analysed areas were manually determined.

### Stool sampling

Patients from the study and control group were asked to collect a stool sample into a screw-capped collection container using a plastic holder to use the collection container in the toilet. The study participants were advised not to use laxatives. The study participants were sampling faeces after overnight fasting. After the stool was collected, patients were delivering the samples within 24 h to our laboratory. The samples were then stored at −80°C until the analyses.

### Short-chain fatty acids

#### Isolation of short-chain fatty acids

Isolation was performed by suspending 0.5 g of a stool sample in a test tube with 2.5 ml of deionised water. The samples were thoroughly mixed on a shaker for 10 minutes. The pH of the samples was then checked and brought to a final pH of 2 to 3 by the addition of 200 μl of 2 M HCl. 36 μl of the internal standard (IS) was added to each sample. The samples were then centrifuged for 10 minutes at 5000 rpm. Next, the obtained supernatant was transferred to vials through a syringe filter (Ø 400 μm) and analysed by gas chromatography.

### Gas chromatography

Gas chromatographic analysis was performed using an Agilent Technologies 1260 A GC chromatograph and a flame ionisation detector (FID) (Agilent Dako, Santa Clara, CA, USA). A fused silica (quartz) capillary column with a free fatty acid phase (DB-FFAP, 30 m × 0.53 mm × 0.5 μm) was used for the analyses. The carrier gas (mobile phase) was hydrogen at a flow rate of 14.4 ml/min. The analysis was done with a temperature gradient, the starting temperature was 100°C and was held for 0.5 minutes. The temperature was then increased by another 8°C and held for 1 minute until the temperature was 180°C. Eventually the temperature was increased to 200°C by increasing it by 20°C for a minute and then keeping it for 5 minutes. The injection volume of one sample was 1 μl, and the analysis time for one sample was 17.5 minutes. The following SCFAs were analysed in the study: acetic acid (C2:0), propionic acid (C3:0), isobutyric acid (C4:0i), butyric acid (C4:0n), isovaleric acid (C5:0i), valeric acid (C5:0n), isocaproic acid (C6:0i), caproic acid (C6:0n), and enanthic acid (C7:0). However, in the analysis of the results, C2:0-C6:0 acids (produced with the participation of the intestinal microflora) were taken into account.

### Statistical analysis

Statistical analysis was performed using the SPSS Statistics 13.0 software (StatSoft, Krakow, Poland). The study sample was described in terms of basic statistics (mean, median, standard deviation (SD), minimum, and maximum values). The normality of the distribution was assessed using the Shapiro-Wilk test. The differences between the groups were determined by the Mann-Whitney *U* test. Spearman’s rank correlation coefficient was applied. The level of significance was set at *p* ≤ 0.05.

## Supplementary Materials

Supplementary Tables 1-2

Supplementary Tables 3-8
