# Supplementary material for: Tissue immunoexpression of IL-6 and IL-18 in aging men with BPH and MetS and their relationship with lipid parameters and gut microbiota-derived short chain fatty acids
Source: Aging (Albany NY). 2023 Oct 16;15(20):10875–96. doi: 10.18632/aging.205091 (PMC10637784; doi:10.18632/aging.205091)
Supplement: Supplementary Tables 1-2 [file aging-15-205091-s001.pdf]

SUPPLEMENTARY TABLES

Supplementary Table 1. Analysis of the location and intensity of IL-6 expression in the cytoplasm of prostate tissue cells from patients with BPH.

Immunoexpression of interleukin 6 (IL-6)												
Location of the reaction	Expression intensification	BPH without MetS					BPH with MetS					*p*-value
		Mean	Median	Min	Max	SD	Mean	Median	Min	Max	SD	
Prostate stromal cells	% of IL-6 (+) cells	79.457	87.932	16.191	100	23.46	90.132	97.126	24.689	100	18.142	**0.034***
	IL-6 (3+)	2.574	0.116	0	53.369	8.565	6.404	1.061	0	54.212	12.43	0.081
	IL-6 (2+)	18.464	7.525	0	96.855	22.753	29.793	23.118	0	80.397	23.338	**0.035***
	IL-6 (1+)	58.419	59.183	0	97.333	22.662	53.935	57.173	0	91.111	26.737	0.676
	IL-6 (−)	20.543	12.068	0	83.81	23.46	9.868	2.874	0	75.311	18.142	**0.034***
Prostate glandular epithelial cells	% of IL -6 (+) cells	74.694	82.642	16.191	100	24.347	77.888	90.568	9.873	100	27.786	0.353
	IL-6 (3+)	2.22	0.17	0	20.315	4.56	3.204	0.965	0	28.117	5.887	0.392
	IL-6 (2+)	19.318	6.704	0	93.848	25.183	23.21	19.482	0	74.415	20.556	0.345
	IL-6 (1+)	53.157	57.476	5.227	90.075	22.042	51.474	53.725	9.747	82.503	20.544	0.771
	IL-6 (−)	25.306	17.358	0	83.81	24.347	22.112	9.432	0	90.127	27.786	0.353

Abbreviations: IL-6: interleukin 6; the intensity of staining: (3+): strong immunoexpression; (2+): moderate immunoexpression; (1+): weak immunoexpression; IL-6 (−): negative cells; MetS: metabolic syndrome; Min: minimum; Max: maximum; SD: standard deviation; p: statistical significance; *: statistical significant parameter.

Supplementary Table 2. Analysis of the location and intensity of IL-18 expression in the cytoplasm of prostate tissue cells from patients with BPH.

Immunoexpression of interleukin 18 (IL-18)												
Location of the reaction	Expression intensification	BPH without MetS					BPH with MetS					*p*-value
		Mean	Median	Min	Max	SD	Mean	Median	Min	Max	SD	
Prostate stromal cells	Percentage of IL-18 (+) cells	76.737	93.494	5.899	100	28.961	90.905	93.467	69.581	99.874	9.619	0.217
	IL-18 (3+)	3.224	0.236	0	64.841	10.078	2.832	0.635	0	19.153	4.361	0.169
	IL-18 (2+)	17.854	10.925	0	55.459	18.549	24.79	18.152	0.269	64.054	20.721	0.075
	IL-18 (1+)	55.658	62.013	2.998	90.236	23.492	63.37	67.848	26.939	91.559	19.137	0.198
	IL-18 (−)	23.263	6.506	0	94.101	28.961	9.095	6.533	0.126	30.419	9.619	0.217
Prostate glandular epithelial cells	Percentage of IL-18 (+) cells	81.942	92.163	20.327	186.229	29.448	95.143	98.06	70.605	100	7.072	0.068
	IL-18 (3+)	15.214	1.976	0	72.167	23.132	23.035	14.779	0.078	64.646	23.612	0.071
	IL-18 (2+)	21.146	21.012	0.023	58.537	17.479	31.131	27.5	4.583	66.864	16.105	**0.040***
	IL-18 (1+)	45.505	47.086	5.882	91.05	22.76	40.977	31.665	8.817	80.321	23.514	0.448
	IL-18 (−)	18.058	9.357	0	79.673	22.823	4.857	1.94	0	29.395	7.072	**0.030***

Abbreviations: IL-18: interleukin 18; the intensity of staining: (3+): strong immunoexpression; (2+): moderate immunoexpression; (1+): weak immunoexpression; IL-18 (−): negative cells; MetS: metabolic syndrome; Min: minimum; Max: maximum; SD: standard deviation; p: statistical significance; *: statistical significant parameter.
