# Supplementary material for: Tissue immunoexpression of IL-6 and IL-18 in aging men with BPH and MetS and their relationship with lipid parameters and gut microbiota-derived short chain fatty acids
Source: Aging (Albany NY). 2023 Oct 16;15(20):10875–96. doi: 10.18632/aging.205091 (PMC10637784; doi:10.18632/aging.205091)
Supplement: Supplementary Tables 3-8 [file aging-15-205091-s002.docx]

**Supplementary Table 3. Correlations between the intensity of staining of IL-6 in stromal and glandular epithelial cells and biochemical parameters in patients with BPH depending on the presence of MetS.**

| **Biochemical parameters** | **Patients with BPH without MetS** | | | | | | | | | | | | | | | | | | | |
| --- | --- | --- | --- | --- | --- | --- | --- | --- | --- | --- | --- | --- | --- | --- | --- | --- | --- | --- | --- | --- |
|  | **Immunoexpression IL-6** | | | | | | | | | | | | | | | | | | | |
|  | **Prostate stromal cells** | | | | | | | | | | **Prostate glandular epithelial cells** | | | | | | | | | |
|  | **Percentage of IL -6 (+) cells** | | **IL-6 (3+)** | | **IL-6 (2+)** | | **IL-6 (1+)** | | **IL-6 (-)** | | **Percentage of IL -6 (+) cells** | | **IL-6 (3+)** | | **IL-6 (2+)** | | **IL-6 (1+)** | | **IL-6 (-)** | |
|  | **R** | **p** | **R** | **p** | **R** | **p** | **R** | **p** | **R** | **p** | **R** | **p** | **R** | **p** | **R** | **p** | **R** | **p** | **R** | **p** |
| **TG**  [mmol/l] | 0.150 | 0.355 | 0.131 | 0.420 | 0.141 | 0.386 | -0.009 | 0.957 | -0.150 | 0.355 | 0.126 | 0.479 | 0.123 | 0.487 | 0.142 | 0.422 | 0.136 | 0.442 | -0.126 | 0.479 |
| **Cholesterol** [mg/dl] | -0.080 | 0.624 | 0.022 | 0.891 | 0.005 | 0.974 | -0.194 | 0.230 | 0.080 | 0.624 | 0.036 | 0.842 | -0.110 | 0.537 | -0.097 | 0.584 | 0.175 | 0.322 | -0.036 | 0.842 |
| **HDL**  [mg/dl] | **-0.312** | **0.050*** | -0.202 | 0.212 | -0.153 | 0.346 | 0.148 | 0.362 | **0.312** | **0.050*** | **-0.400** | **0.019*** | -0.330 | 0.057 | **-0.404** | **0.018*** | 0.239 | 0.174 | **0.400** | **0.019*** |
| **LDL**  [mg/dl] | -0.056 | 0.731 | 0.028 | 0.866 | -0.009 | 0.958 | -0.224 | 0.164 | 0.056 | 0.731 | 0.087 | 0.626 | -0.052 | 0.771 | -0.036 | 0.840 | 0.070 | 0.696 | -0.087 | 0.626 |
| **Glucose** [mg/dl] | -0.091 | 0.575 | -0.213 | 0.187 | -0.123 | 0.449 | 0.086 | 0.599 | 0.091 | 0.575 | -0.139 | 0.432 | -0.229 | 0.192 | -0.147 | 0.407 | -0.034 | 0.850 | 0.139 | 0.432 |
| **Biochemical parameters** | **Patients with BPH with MetS** | | | | | | | | | | | | | | | | | | | |
|  | **Immunoexpression IL-6** | | | | | | | | | | | | | | | | | | | |
|  | **Prostate stromal cells** | | | | | | | | | | **Prostate glandular epithelial cells** | | | | | | | | | |
|  | **Percentage of IL -6 (+) cells** | | **IL-6 (3+)** | | **IL-6 (2+)** | | **IL-6 (1+)** | | **IL-6 (-)** | | **Percentage of IL -6 (+) cells** | | **IL-6 (3+)** | | **IL-6 (2+)** | | **IL-6 (1+)** | | **IL-6 (-)** | |
|  | **R** | **p** | **R** | **p** | **R** | **p** | **R** | **p** | **R** | **p** | **R** | **p** | **R** | **p** | **R** | **p** | **R** | **p** | **R** | **p** |
| **TG**  [mmol/l] | 0.071 | 0.692 | 0.102 | 0.567 | -0.020 | 0.911 | 0.021 | 0.906 | -0.071 | 0.692 | -0.140 | 0.486 | -0.050 | 0.805 | -0.181 | 0.365 | 0.075 | 0.712 | 0.140 | 0.486 |
| **Cholesterol** [mg/dl] | 0.040 | 0.824 | 0.185 | 0.295 | 0.050 | 0.780 | -0.095 | 0.595 | -0.040 | 0.824 | -0.050 | 0.804 | 0.042 | 0.835 | -0.027 | 0.892 | -0.205 | 0.306 | 0.050 | 0.804 |
| **HDL**  [mg/dl] | 0.214 | 0.225 | **0.341** | **0.049*** | **0.379** | **0.027*** | -0.224 | 0.202 | -0.214 | 0.225 | 0.142 | 0.481 | 0.156 | 0.438 | 0.235 | 0.238 | 0.016 | 0.939 | -0.142 | 0.481 |
| **LDL**  [mg/dl] | 0.067 | 0.708 | 0.011 | 0.951 | -0.052 | 0.769 | -0.033 | 0.855 | -0.067 | 0.708 | -0.002 | 0.990 | 0.074 | 0.713 | 0.009 | 0.966 | -0.248 | 0.213 | 0.002 | 0.990 |
| **Glucose**  [mg/dl] | -0.142 | 0.422 | 0.078 | 0.663 | -0.024 | 0.893 | -0.102 | 0.566 | 0.142 | 0.422 | -0.380 | 0.051 | -0.156 | 0.437 | -0.273 | 0.168 | 0.037 | 0.855 | 0.380 | 0.051 |
| **BPH** – benign prostatic hyperplasia; **MetS** – metabolic syndrome; **IL-6** – interleukin 6; the intensity of staining: **(3+)** – strong immunoexpression; **(2+) –** moderate immunoexpression; **(1+)** – weak immunoexpression; **IL- (-)** – negative cells; **TG** – triglycerides; **HDL** – high density lipoprotein; **LDL** – low density lipoprotein; **R** - correlation coefficient; **p** – statistical significance; ***** - statistical significant parameter | | | | | | | | | | | | | | | | | | | | |

**Supplementary Table 4. Correlations between the intensity of staining of IL-18 in stromal and glandular epithelial cells and biochemical parameters in patients with BPH depending on the presence of MetS.**

| **Biochemical parameters** | **Patients with BPH without MetS** | | | | | | | | | | | | | | | | | | | | | | |
| --- | --- | --- | --- | --- | --- | --- | --- | --- | --- | --- | --- | --- | --- | --- | --- | --- | --- | --- | --- | --- | --- | --- | --- |
|  | **Immunoexpression of IL-18** | | | | | | | | | | | | | | | | | | | | | | |
|  | **Prostate stromal cells** | | | | | | | | | | | | **Prostate glandular epithelial cells** | | | | | | | | | | |
|  | **Percentage of IL-18 (+) cells** | | **IL-18 (3+)** | | **IL-18 (2+)** | | | **IL-18 (1+)** | | | **IL-18 (-)** | | **Percentage of IL-18 (+) cells** | | **IL-18 (3+)** | | **IL-18 (2+)** | | **IL-18 (1+)** | | | **IL-18 (-)** | |
|  | **R** | **p** | **R** | **p** | **R** | **p** | | **R** | **p** | | **R** | **p** | **R** | **p** | **R** | **p** | **R** | **p** | **R** | | **p** | **R** | **p** |
| **TG**  [mmol/l] | **0.396** | **0.007*** | **0.496** | **0.001*** | **0.436** | **0.003*** | | -0.082 | 0.594 | | **-0.400** | **0.006*** | 0.324 | 0.070 | 0.206 | 0.276 | 0.269 | 0.136 | -0.242 | | 0.182 | -0.291 | 0.106 |
| **Cholesterol** [mg/dl] | 0.119 | 0.435 | 0.215 | 0.156 | 0.127 | 0.405 | | -0.010 | 0.950 | | -0.122 | 0.424 | 0.077 | 0.675 | -0.081 | 0.672 | 0.199 | 0.274 | -0.216 | | 0.234 | -0.100 | 0.586 |
| **HDL**  [mg/dl] | **-0.362** | **0.015*** | -0.256 | 0.090 | **-0.377** | **0.011*** | | -0.107 | 0.483 | | **0.357** | **0.016*** | **-0.376** | **0.034*** | -0.340 | 0.066 | -0.339 | 0.058 | **0.414** | | **0.018*** | 0.330 | 0.065 |
| **LDL**  [mg/dl] | 0.075 | 0.625 | 0.123 | 0.422 | 0.115 | 0.450 | | 0.100 | 0.515 | | -0.068 | 0.656 | 0.151 | 0.408 | 0.001 | 0.996 | 0.241 | 0.184 | -0.300 | | 0.095 | -0.164 | 0.370 |
| **Glucose** [mg/dl] | -0.225 | 0.138 | -0.233 | 0.124 | -0.226 | 0.136 | | 0.172 | 0.257 | | 0.228 | 0.131 | -0.128 | 0.485 | -0.338 | 0.068 | -0.158 | 0.389 | 0.096 | | 0.601 | 0.075 | 0.682 |
| **Biochemical parameters** | **Patients with BPH with MetS** | | | | | | | | | | | | | | | | | | | | | | |
|  | **Immunoexpression of IL-18** | | | | | | | | | | | | | | | | | | | | | | |
|  | **Prostate stromal cells** | | | | | | | | | | | | **Prostate glandular epithelial cells** | | | | | | | | | | |
|  | **Percentage of IL-18 (+) cells** | | **IL-18 (3+)** | | **IL-18 (2+)** | | **IL-18 (1+)** | | | **IL-18 (-)** | | | **Percentage of IL-18 (+) cells** | | **IL-18 (3+)** | | **IL-18 (2+)** | | | **IL-18 (1+)** | | **IL-18 (-)** | |
|  | **R** | **p** | **R** | **p** | **R** | **p** | | **R** | **p** | | **R** | **p** | **R** | **p** | **R** | **p** | **R** | **p** | **R** | | **p** | **R** | **p** |
| **TG**  [mmol/l] | 0.088 | 0.633 | 0.095 | 0.613 | 0.199 | 0.274 | | -0.301 | 0.094 | | -0.088 | 0.633 | 0.263 | 0.225 | 0.378 | 0.075 | 0.139 | 0.527 | **-0.505** | | **0.014*** | -0.263 | 0.225 |
| **Cholesterol** [mg/dl] | 0.070 | 0.702 | 0.040 | 0.831 | 0.093 | 0.611 | | -0.070 | 0.702 | | -0.070 | 0.702 | 0.069 | 0.754 | 0.185 | 0.399 | -0.028 | 0.900 | -0.217 | | 0.319 | -0.069 | 0.754 |
| **HDL**  [mg/dl] | -0.189 | 0.300 | -0.106 | 0.569 | -0.152 | 0.405 | | 0.202 | 0.267 | | 0.189 | 0.300 | -0.259 | 0.233 | **-0.454** | **0.029*** | 0.054 | 0.807 | **0.512** | | **0.013*** | 0.259 | 0.233 |
| **LDL**  [mg/dl] | 0.094 | 0.609 | 0.071 | 0.703 | 0.095 | 0.604 | | -0.011 | 0.952 | | -0.094 | 0.609 | -0.053 | 0.809 | 0.206 | 0.347 | -0.258 | 0.235 | -0.099 | | 0.654 | 0.053 | 0.809 |
| **Glucose** [mg/dl] | -0.282 | 0.118 | -0.226 | 0.222 | -0.248 | 0.171 | | 0.070 | 0.702 | | 0.282 | 0.118 | -0.296 | 0.170 | **-0.416** | **0.049*** | -0.072 | 0.745 | 0.297 | | 0.169 | 0.296 | 0.170 |
| **BPH** – benign prostatic hyperplasia; **MetS** – metabolic syndrome; **IL-18** – interleukin 18; the intensity of staining: **(3+)** – strong immunoexpression; **(2+) –**  moderate immunoexpression; **(1+)** – weak immunoexpression; **IL- (-)** – negative cells; **TG** – triglycerides; **HDL** – high density lipoprotein; **LDL** – low density lipoprotein; **R** - correlation coefficient; **p** – statistical significance; ***** - statistical significant parameter | | | | | | | | | | | | | | | | | | | | | | | |

**Supplementary Table 5. Correlations between the intensity of staining of IL-6 in stromal and glandular epithelial cells and TG/HDL and LDL/HDL ratio in patients with BPH depending on the presence of MetS.**

| **Biochemical parameters** | **Patients with BPH without MetS** | | | | | | | | | | | | | | | | | | | | |
| --- | --- | --- | --- | --- | --- | --- | --- | --- | --- | --- | --- | --- | --- | --- | --- | --- | --- | --- | --- | --- | --- |
|  | **Immunoexpression of IL-6** | | | | | | | | | | | | | | | | | | | | |
|  | **Prostate stromal cells** | | | | | | | | | | **Prostate glandular epithelial cells** | | | | | | | | | | |
|  | **Percentage of IL-6 (+) cells** | | **IL-6 (3+)** | | **IL-6 (2+)** | | **IL-6 (1+)** | | **IL-6 (-)** | | **Percentage of IL-6 (+) cells** | | **IL-6 (3+)** | | **IL-6 (2+)** | | **IL-6 (1+)** | | | **IL-6 (-)** | |
|  | **R** | **p** | **R** | **p** | **R** | **p** | **R** | **p** | **R** | **p** | **R** | **p** | **R** | **p** | **R** | **p** | **R** | | **p** | **R** | **p** |
| **TG/HDL** | 0,251 | 0,118 | 0,198 | 0,220 | 0,155 | 0,340 | -0,048 | 0,769 | -0,251 | 0,118 | 0,259 | 0,139 | 0,263 | 0,134 | 0,278 | 0,111 | 0,031 | | 0,863 | -0,259 | 0,139 |
| **LDL/HDL** | 0,054 | 0,739 | 0,086 | 0,598 | 0,044 | 0,788 | -0,220 | 0,173 | -0,054 | 0,739 | 0,211 | 0,231 | 0,076 | 0,671 | 0,123 | 0,487 | -0,039 | | 0,827 | -0,211 | 0,231 |
| **Biochemical parameters** | **Patients with BPH with MetS** | | | | | | | | | | | | | | | | | | | | |
|  | **Immunoexpression of IL-6** | | | | | | | | | | | | | | | | | | | | |
|  | **Prostate stromal cells** | | | | | | | | | | **Prostate glandular epithelial cells** | | | | | | | | | | |
|  | **Percentage of IL-6 (+) cells** | | **IL-6 (3+)** | | **IL-6 (2+)** | | **IL-6 (1+)** | | **IL-6 (-)** | | **Percentage of IL-6 (+) cells** | | **IL-6 (3+)** | | **IL-6 (2+)** | | | **IL-6 (1+)** | | **IL-6 (-)** | |
|  | **R** | **p** | **R** | **p** | **R** | **p** | **R** | **p** | **R** | **p** | **R** | **p** | **R** | **p** | **R** | **p** | **R** | | **p** | **R** | **p** |
| **TG/HDL** | -0,068 | 0,704 | -0,042 | 0,812 | -0,179 | 0,312 | 0,061 | 0,731 | 0,068 | 0,704 | -0,263 | 0,185 | -0,181 | 0,365 | -0,313 | 0,112 | 0,045 | | 0,825 | 0,263 | 0,185 |
| **LDL/HDL** | -0,011 | 0,950 | -0,115 | 0,518 | -0,211 | 0,231 | 0,080 | 0,655 | 0,011 | 0,950 | -0,046 | 0,818 | -0,048 | 0,810 | -0,134 | 0,506 | -0,158 | | 0,431 | 0,046 | 0,818 |
| **BPH** – benign prostatic hyperplasia; **MetS** – metabolic syndrome; **IL-6** – interleukin 6; the intensity of staining: **(3+)** – strong immunoexpression; **(2+) –**  moderate immunoexpression; **(1+)** – weak immunoexpression; **IL- (-)** – negative cells; **TG** – triglycerides; **HDL** – high density lipoprotein; **LDL** – low density lipoprotein; **R** - correlation coefficient; **p** – statistical significance; ***** - statistical significant parameter | | | | | | | | | | | | | | | | | | | | | |

**Supplementary Table 6. Correlations between the intensity of staining of IL-18 in stromal and glandular epithelial cells and TG/HDL and LDL/HDL ratio in patients with BPH depending on the presence of MetS.**

| **Biochemical parameters** | **Patients with BPH without MetS** | | | | | | | | | | | | | | | | | | | | | | |
| --- | --- | --- | --- | --- | --- | --- | --- | --- | --- | --- | --- | --- | --- | --- | --- | --- | --- | --- | --- | --- | --- | --- | --- |
|  | **Immunoexpression of IL-18** | | | | | | | | | | | | | | | | | | | | | | |
|  | **Prostate stromal cells** | | | | | | | | | | | | **Prostate glandular epithelial cells** | | | | | | | | | | |
|  | **Percentage of IL-18 (+) cells** | | **IL-18 (3+)** | | **IL-18 (2+)** | | | **IL-18 (1+)** | | | **IL-18 (-)** | | **Percentage of IL-18 (+) cells** | | **IL-18 (3+)** | | **IL-18 (2+)** | | **IL-18 (1+)** | | | **IL-18 (-)** | |
|  | **R** | **p** | **R** | **p** | **R** | **p** | | **R** | **p** | | **R** | **p** | **R** | **p** | **R** | **p** | **R** | **p** | **R** | | **p** | **R** | **p** |
| **TG/HDL** | 0,078 | 0,670 | 0,064 | 0,733 | 0,157 | 0,391 | | -0,276 | 0,127 | | -0,078 | 0,670 | 0,269 | 0,215 | 0,374 | 0,079 | 0,075 | 0,733 | **-0,500** | | **0,015*** | -0,269 | 0,215 |
| **LDL/HDL** | 0,186 | 0,222 | 0,198 | 0,192 | 0,228 | 0,132 | | 0,099 | 0,516 | | -0,178 | 0,243 | 0,275 | 0,127 | 0,117 | 0,538 | 0,302 | 0,093 | **-0,375** | | **0,034*** | -0,245 | 0,176 |
| **Biochemical parameters** | **Patients with BPH with MetS** | | | | | | | | | | | | | | | | | | | | | | |
|  | **Immunoexpression of IL-18** | | | | | | | | | | | | | | | | | | | | | | |
|  | **Prostate stromal cells** | | | | | | | | | | | | **Prostate glandular epithelial cells** | | | | | | | | | | |
|  | **Percentage of IL-18 (+) cells** | | **IL-18 (3+)** | | **IL-18 (2+)** | | **IL-18 (1+)** | | | **IL-18 (-)** | | | **Percentage of IL-18 (+) cells** | | **IL-18 (3+)** | | **IL-18 (2+)** | | | **IL-18 (1+)** | | **IL-18 (-)** | |
|  | **R** | **p** | **R** | **p** | **R** | **p** | | **R** | **p** | | **R** | **p** | **R** | **p** | **R** | **p** | **R** | **p** | **R** | | **p** | **R** | **p** |
| **TG/HDL** | **0,439** | **0,003*** | **0,466** | **0,001*** | **0,459** | **0,002*** | | -0,019 | 0,904 | | **-0,442** | **0,002*** | **0,399** | **0,024*** | 0,268 | 0,153 | **0,366** | **0,039*** | -0,332 | | 0,063 | -0,337 | 0,059 |
| **LDL/HDL** | 0,192 | 0,291 | 0,130 | 0,485 | 0,177 | 0,331 | | -0,122 | 0,506 | | -0,192 | 0,291 | 0,082 | 0,710 | **0,433** | **0,039*** | -0,243 | 0,264 | -0,357 | | 0,095 | -0,082 | 0,710 |
| **BPH** – benign prostatic hyperplasia; **MetS** – metabolic syndrome; **IL-18** – interleukin 18; the intensity of staining: **(3+)** – strong immunoexpression; **(2+) –**  moderate immunoexpression; **(1+)** – weak immunoexpression; **IL- (-)** – negative cells; **TG** – triglycerides; **HDL** – high density lipoprotein; **LDL** – low density lipoprotein; **R** - correlation coefficient; **p** – statistical significance; ***** - statistical significant parameter | | | | | | | | | | | | | | | | | | | | | | | |

**Supplementary Table 7. Correlations between the analyzed short-chain fatty acids and the percentage of IL-6 (+) cells in stromal cells and prostate glandular epithelial cells in BPH patients depending on the coexistence of the metabolic syndrome.**

| **Patients with BPH without MetS** | | | | | | | | | | | | | | | | |
| --- | --- | --- | --- | --- | --- | --- | --- | --- | --- | --- | --- | --- | --- | --- | --- | --- |
|  | **Prostate stromal cells** | | | | | | | | **Prostate glandular epithelial cells** | | | | | | | |
| **SCFAs (%)** | **Percentage of IL-6 (+) cells** | | **IL-6 (3+)** | | **IL-6 (2+)** | | **IL-6 (1+)** | | **Percentage of IL-6 (+) cells** | | **IL-6 (3+)** | | **IL-6 (2+)** | | **IL-6 (1+)** | |
|  | R | p | R | p | R | p | R | p | R | p | R | p | R | p | R | p |
| **C 2:0** | 0.141 | 0.387 | -0.025 | 0.879 | 0.053 | 0.746 | 0.204 | 0.206 | 0.207 | 0.241 | 0.132 | 0.458 | 0.126 | 0.478 | 0.059 | 0.738 |
| **C 3:0** | -0.017 | 0.916 | -0.238 | 0.140 | -0.202 | 0.212 | 0.190 | 0.239 | 0.042 | 0.812 | -0.154 | 0.386 | -0.160 | 0.367 | 0.080 | 0.653 |
| **C 4:0 i** | 0.097 | 0.553 | 0.189 | 0.242 | 0.223 | 0.167 | -0.143 | 0.378 | 0.191 | 0.280 | 0.054 | 0.762 | 0.188 | 0.286 | 0.080 | 0.655 |
| **C 4:0 n** | -0.122 | 0.455 | -0.007 | 0.964 | -0.097 | 0.553 | -0.029 | 0.860 | -0.158 | 0.373 | -0.038 | 0.830 | -0.083 | 0.642 | -0.114 | 0.519 |
| **C 5:0 i** | 0.102 | 0.532 | 0.191 | 0.239 | 0.213 | 0.188 | -0.155 | 0.339 | 0.180 | 0.310 | 0.058 | 0.744 | 0.170 | 0.337 | 0.087 | 0.624 |
| **C 5:0 n** | -0.206 | 0.201 | -0.072 | 0.660 | -0.078 | 0.632 | -0.257 | 0.109 | -0.317 | 0.068 | -0.125 | 0.480 | -0.102 | 0.565 | -0.213 | 0.226 |
| **C 6:0 i** | -0.019 | 0.907 | -0.003 | 0.988 | 0.000 | 0.998 | -0.056 | 0.729 | -0.046 | 0.796 | 0.120 | 0.497 | 0.136 | 0.443 | -0.164 | 0.355 |
| **C 6:0 n** | -0.271 | 0.091 | -0.152 | 0.350 | -0.148 | 0.363 | -0.173 | 0.286 | **-0.397** | **0.020*** | -0.180 | 0.309 | -0.138 | 0.437 | -0.289 | 0.098 |
| **Patients with BPH with MetS** | | | | | | | | | | | | | | | | |
|  | **Prostate stromal cells** | | | | | | | | **Prostate glandular epithelial cells** | | | | | | | |
| **SCFAs (%)** | **Percentage of IL-6 (+) cells** | | **IL-6 (3+)** | | **IL-6 (2+)** | | **IL-6 (1+)** | | **Percentage of IL-6 (+) cells** | | **IL-6 (3+)** | | **IL-6 (2+)** | | **IL-6 (1+)** | |
|  | R | p | R | p | R | p | R | p | R | p | R | p | R | p | R | p |
| **C 2:0** | 0.117 | 0.511 | -0.139 | 0.433 | -0.084 | 0.635 | **0.381** | **0.026*** | 0.102 | 0.613 | -0.010 | 0.961 | -0.033 | 0.870 | **0.504** | **0.007*** |
| **C 3:0** | 0.072 | 0.687 | 0.038 | 0.831 | 0.025 | 0.890 | 0.172 | 0.331 | -0.189 | 0.344 | -0.010 | 0.961 | -0.093 | 0.643 | 0.316 | 0.109 |
| **C 4:0 i** | -0.175 | 0.321 | -0.187 | 0.290 | -0.169 | 0.340 | -0.071 | 0.690 | 0.160 | 0.425 | -0.002 | 0.994 | -0.062 | 0.760 | 0.121 | 0.548 |
| **C 4:0 n** | -0.021 | 0.907 | 0.240 | 0.171 | 0.204 | 0.247 | **-0.377** | **0.028*** | -0.093 | 0.643 | 0.147 | 0.463 | 0.139 | 0.489 | **-0.643** | **<0.001*** |
| **C 5:0 i** | -0.128 | 0.472 | -0.172 | 0.330 | -0.164 | 0.355 | -0.012 | 0.945 | 0.214 | 0.283 | 0.051 | 0.802 | -0.026 | 0.899 | 0.161 | 0.422 |
| **C 5:0 n** | -0.236 | 0.179 | -0.152 | 0.390 | -0.150 | 0.397 | -0.091 | 0.609 | -0.027 | 0.892 | -0.053 | 0.793 | -0.104 | 0.606 | -0.148 | 0.460 |
| **C 6:0 i** | 0.224 | 0.203 | 0.104 | 0.560 | 0.082 | 0.643 | -0.077 | 0.663 | 0.141 | 0.483 | 0.131 | 0.514 | -0.023 | 0.909 | 0.135 | 0.502 |
| **C 6:0 n** | -0.038 | 0.829 | 0.011 | 0.951 | 0.007 | 0.968 | -0.066 | 0.710 | 0.141 | 0.483 | 0.330 | 0.093 | 0.197 | 0.324 | -0.377 | 0.053 |
| **BPH** – benign prostatic hyperplasia; **MetS** – metabolic syndrome;**IL-6** – interleukin 6; the intensity of staining: **(3+)** – strong immunoexpression; **(2+) –**  moderate immunoexpression; **(1+)** – weak immunoexpression; **SCFAs** – short-chain fatty acids; **C2:0** - acetic acid; **C3:0** - propionic acid; **C4:0i** - isobutyric acid; **C4:0n** - butyric acid; **C5:0i** - isovaleric acid; **C5:0n** - valeric acid; **C6:0i** - isocaproic acid; **C 6:0n** - caproic acid; **R** - correlation coefficient; **p** – statistical significance; ***** - statistical significant parameter | | | | | | | | | | | | | | | | |

**Supplementary Table 8 Correlations between the analyzed short-chain fatty acids and the percentage of IL-18 (+) cells in stromal cells and prostate glandular epithelial cells in BPH patients depending on the coexistence of the metabolic syndrome.**

| **Patients with BPH without MetS** | | | | | | | | | | | | | | | | |
| --- | --- | --- | --- | --- | --- | --- | --- | --- | --- | --- | --- | --- | --- | --- | --- | --- |
|  | **Prostate stromal cells** | | | | | | | | **Prostate glandular epithelial cells** | | | | | | | |
| **SCFAs (%)** | **Percentage of IL-18 (+) cells** | | **IL-18 (3+)** | | **IL-18 (2+)** | | **IL-18 (1+)** | | **Percentage of IL-18 (+) cells** | | **IL-18 (3+)** | | **IL-18 (2+)** | | **IL-18 (1+)** | |
|  | R | p | R | p | R | p | R | p | R | p | R | p | R | p | R | p |
| **C 2:0** | 0.111 | 0.467 | 0.184 | 0.225 | 0.120 | 0.433 | -0.115 | 0.451 | -0.078 | 0.672 | 0.071 | 0.709 | -0.304 | 0.091 | -0.217 | 0.234 |
| **C 3:0** | -0.209 | 0.167 | -0.098 | 0.521 | -0.141 | 0.357 | -0.246 | 0.104 | -0.017 | 0.927 | -0.067 | 0.725 | -0.184 | 0.314 | -0.206 | 0.259 |
| **C 4:0 i** | 0.059 | 0.699 | -0.005 | 0.972 | -0.018 | 0.908 | 0.084 | 0.584 | -0.071 | 0.699 | -0.116 | 0.541 | 0.152 | 0.406 | 0.325 | 0.069 |
| **C 4:0 n** | -0.029 | 0.851 | -0.049 | 0.748 | 0.010 | 0.950 | 0.040 | 0.793 | -0.026 | 0.888 | -0.065 | 0.731 | 0.085 | 0.642 | -0.005 | 0.976 |
| **C 5:0 i** | 0.173 | 0.257 | 0.087 | 0.571 | 0.060 | 0.694 | 0.056 | 0.714 | 0.064 | 0.727 | 0.069 | 0.719 | 0.168 | 0.359 | 0.166 | 0.363 |
| **C 5:0 n** | -0.060 | 0.697 | -0.148 | 0.331 | -0.107 | 0.482 | 0.284 | 0.059 | 0.250 | 0.168 | 0.092 | 0.630 | 0.274 | 0.129 | 0.325 | 0.070 |
| **C 6:0 i** | 0.261 | 0.084 | 0.249 | 0.099 | 0.260 | 0.084 | 0.026 | 0.867 | 0.276 | 0.126 | **0.421** | **0.021*** | 0.265 | 0.143 | -0.092 | 0.616 |
| **C 6:0 n** | 0.041 | 0.791 | -0.122 | 0.424 | 0.017 | 0.909 | 0.180 | 0.238 | 0.123 | 0.503 | 0.109 | 0.566 | 0.209 | 0.252 | 0.256 | 0.157 |
| **Patients with BPH with MetS** | | | | | | | | | | | | | | | | |
|  | **Prostate stromal cells** | | | | | | | | **Prostate glandular epithelial cells** | | | | | | | |
| **SCFAs (%)** | **Percentage of IL-18 (+) cells** | | **IL-18 (3+)** | | **IL-18 (2+)** | | **IL-18 (1+)** | | **Percentage of IL-18 (+) cells** | | **IL-18 (3+)** | | **IL-18 (2+)** | | **IL-18 (1+)** | |
|  | R | p | R | p | R | p | R | p | R | p | R | p | R | p | R | p |
| **C 2:0** | -0.202 | 0.268 | 0.119 | 0.525 | -0.081 | 0.659 | -0.051 | 0.782 | **-0.424** | **0.044*** | -0.251 | 0.248 | -0.245 | 0.260 | 0.246 | 0.258 |
| **C 3:0** | -0.149 | 0.416 | -0.035 | 0.852 | -0.084 | 0.646 | -0.098 | 0.594 | -0.049 | 0.823 | -0.074 | 0.737 | -0.034 | 0.879 | -0.127 | 0.562 |
| **C 4:0 i** | 0.012 | 0.948 | 0.061 | 0.745 | 0.011 | 0.952 | -0.078 | 0.671 | 0.015 | 0.946 | -0.130 | 0.553 | 0.188 | 0.391 | 0.048 | 0.826 |
| **C 4:0 n** | 0.170 | 0.351 | -0.142 | 0.447 | 0.059 | 0.747 | 0.136 | 0.458 | 0.392 | 0.064 | 0.199 | 0.364 | 0.168 | 0.444 | -0.165 | 0.452 |
| **C 5:0 i** | -0.052 | 0.779 | -0.002 | 0.991 | -0.061 | 0.741 | -0.034 | 0.851 | 0.090 | 0.683 | -0.037 | 0.868 | -0.159 | 0.468 | 0.112 | 0.612 |
| **C 5:0 n** | 0.100 | 0.586 | -0.008 | 0.966 | 0.018 | 0.922 | 0.012 | 0.948 | 0.095 | 0.667 | 0.142 | 0.517 | 0.034 | 0.879 | -0.098 | 0.657 |
| **C 6:0 i** | 0.253 | 0.162 | 0.344 | 0.058 | 0.310 | 0.084 | -0.276 | 0.126 | 0.337 | 0.116 | 0.290 | 0.180 | 0.209 | 0.337 | -0.371 | 0.082 |
| **C 6:0 n** | 0.255 | 0.159 | 0.224 | 0.225 | 0.319 | 0.076 | -0.293 | 0.104 | 0.194 | 0.376 | 0.176 | 0.422 | -0.014 | 0.950 | -0.171 | 0.435 |
| **BPH** – benign prostatic hyperplasia; **MetS** – metabolic syndrome; **IL-18** – interleukin 18; the intensity of staining: **(3+)** – strong immunoexpression; **(2+) –** moderate immunoexpression; **(1+)** – weak immunoexpression; **SCFAs** – short-chain fatty acids; **C2:0** - acetic acid; **C3:0** - propionic acid; **C4:0i** - isobutyric acid; **C4:0n** - butyric acid; **C5:0i** - isovaleric acid; **C5:0n** - valeric acid; **C6:0i** - isocaproic acid; **C 6:0n** - caproic acid; **R** - correlation coefficient; **p** – statistical significance; ***** - statistical significant parameter | | | | | | | | | | | | | | | | |
